# FOS Knockdown Alleviates *Helicobacter pylori*‐Infected Gastritis by Suppressing Mast Cell Activation and Treg Polarization

**DOI:** 10.1155/mi/4596288

**Published:** 2026-03-23

**Authors:** Wen Ma, Ruidong Han, Lei Wang

**Affiliations:** ^1^ Department of Gastrointestinal Surgery, General Hospital Of Ningxia Medical University, 804 Shengli Street, Xingqing Area, Yinchuan, 750003, Ningxia, China, nxmu.edu.cn

**Keywords:** fos proto-oncogene, gastritis, *Helicobacter pylori*, machine learning, mast cells, regulatory t cells

## Abstract

**Background:**

*Helicobacter pylori* (HP) is a major cause of gastritis, yet the epithelial mechanisms linking infection‐induced stress to mast cell and Treg responses remain poorly defined.

**Methods:**

Three datasets (GSE5081, GSE27411, and GSE233973) were integrated and analyzed using weighted gene co‐expression network analysis (WGCNA) and machine learning algorithms. Mast cell‐related hub gene expressions were evaluated with quantitative real‐time polymerase chain reaction (qRT‐PCR), and inflammatory cytokines were quantified using the enzyme‐linked immunosorbent assay (ELISA). Histopathological changes were evaluated using hematoxylin and eosin (HE), Giemsa, and Warthin‐Starry silver staining. Cell apoptosis was assessed by flow cytometry, and mast cell and Treg cell activities were analyzed by Transwell assays, histamine detection, and immunohistochemistry (IHC).

**Results:**

Fos proto‐oncogene (FOS), ribonucleotide reductase regulatory subunit M2 (RRM2), and RAD51 recombinase (RAD51) were identified as mast cell‐related hub genes, all of which were upregulated in HP‐induced gastritis mice. In vitro, HP infection or CagA stimulation increased FOS expression in gastric epithelial cells. FOS knockdown in HP‐infected mice alleviated gastric mucosal injury, reduced bacterial burden, and decreased pro‐inflammatory cytokine levels. FOS silencing enhanced GES‐1 cell viability and suppressed apoptosis. In HP‐infected GES‐1 cells, FOS silencing inhibited mast cell migration, cytokine secretion, including C–C motif chemokine ligand 2 (CCL2), interleukin‐33 (IL‐33), and stem cell factor (SCF), as well as histamine release, accompanied by reduced Treg polarization and decreased expression of transforming growth factor‐β and forkhead box P3.

**Conclusion:**

FOS silencing inhibited mast cell activation and Treg cell polarization in HP‐induced gastritis, suggesting its promising value as an intervention point in HP‐driven gastritis.

## 1. Introduction

Gastritis is among the most prevalent gastrointestinal disorders globally, and persistent gastric mucosal inflammation results in mucosal atrophy, intestinal metaplasia, and even progression to gastric cancer [1]. Chronic gastritis is a multifactorial disease, with *Helicobacter pylori* (HP) infection being one of its primary etiological factors [2]. HP infection remains a significant worldwide public health concern, affecting ~43.1% of the population [3]. Increased density and deeper colonization of HP are associated with heightened gastritis activity and severity, and a higher risk of ulcer development [4]. Standard triple, bismuth‐containing quadruple, and sequential regimens are presently advised for the eradication of HP; however, increasing antibiotic resistance has markedly reduced their success rates [5, 6]. Therefore, further elucidating the molecular mechanisms underlying HP‐induced gastritis is essential for identifying novel therapeutic targets and improving therapeutic efficacy.

Although the innate immune system is highly effective, accumulating evidence suggests that HP employs sophisticated strategies to evade host immunity, facilitating persistent infection and promoting neoplastic transformation [7, 8]. HP modulates pathogen‐associated molecular patterns to attenuate Toll‐like receptor signaling, impairs antigen presentation, induces regulatory T cells (Tregs), and upregulates immune checkpoints such as programmed death‐ligand 1 (PD‐L1), thereby facilitating chronic infection and persistent inflammation [9]. Mast cells are a unique population of immune cells involved in Immunoglobulin E (IgE)‐mediated responses, modulation of chronic inflammation, and induction of Treg cells [10, 11]. During HP infection, Hypoxia‐inducible factor‐1α (HIF‐1α)‐associated G protein‐coupled receptor 171 (GPR171) facilitates mast cell‐derived C–C motif chemokine ligand 2 (CCL2) secretion, contributing to gastric inflammatory responses [12]. Tryptase‐positive mast cells are found to co‐localize with HP in gastric biopsy specimens, suggesting their close involvement in gastric mucosal inflammation [13]. However, the mechanistic role of mast cells in HP‐induced gastritis remains largely unclear, particularly regarding their interaction with Tregs.

Fos proto‐oncogene (FOS), a core subunit of the activator protein‐1 (AP‐1) transcription factor complex, is critically involved in the regulation of cell proliferation, differentiation, programmed cell death, and inflammatory responses [14, 15]. Recent studies have implicated FOS in immune regulation through modulating the activation and function of various immune cells, such as neutrophils and macrophages [16, 17]. Additionally, Wang et al. [18] have found that suppressing cellular‐Fos (c‐Fos) expression reduces IgE‐associated mast cell activation and allergic inflammation by disrupting the repressive AP‐1/early growth response 1 (Egr1)/interleukin‐4 (IL‐4) signaling axis. Moreover, FOS is identified as a key gene associated with chronic atrophic gastritis (CAG) and a potential therapeutic target [19]. By blocking the phosphorylation of c‐Fos, quercetin suppresses tumor necrosis factor‐alpha (TNF‐α)‐stimulated upregulation of matrix metalloproteinase‐9 (MMP‐9), thereby exerting anti‐inflammatory effects in gastric epithelial GES‐1 cells [20]. Nonetheless, the role of FOS in HP‐induced gastritis remains largely unexplored. particularly in the mast cell‐associated immune modulation.

This study sought to uncover critical regulators involved in mast cell‐associated immune responses during HP infection. We identified FOS as a potential hub gene by bioinformatics approaches. We further found that FOS knockdown alleviated gastric mucosal injury, suppressed proinflammatory cytokine release, and reduced mast cell activation and Treg polarization. These results shed light on a novel mechanism by which HP manipulates host immunity via the epithelial‐mast cell‐Treg axis.

## 2. Materials and Methods

### 2.1. Data Acquisition and Preprocessing

Gene expression profiles were obtained from the Gene Expression Omnibus (GEO) database (https://www.ncbi.nlm.nih.gov/geo/). Datasets related to “Gastritis,” “Helicobacter,” “*Homo sapiens*,” and “array” were searched, and three microarray datasets, including GSE5081, GSE27411, and GSE233973, were included in subsequent analyses. Gene expression features and samples exhibiting over 50% missing data were removed from subsequent analyses. Residual missing entries were imputed via the k‐nearest neighbors algorithm (impute.knn function, impute R package), with the optimal number of neighbors (K) determined by local estimation. The datasets were then normalized using a log_2_(*X* + 1) transformation. The processed expression matrices were merged by the inSilicoMerging package, followed by Empirical Bayes‐based batch correction. The effectiveness of batch correction was assessed using density plots and Uniform Manifold Approximation and Projection (UMAP) through the umap and ggplot2 packages. Principal component analysis (PCA) was employed to assess data variance and clustering using the FactoMineR package. Two‐ and three‐dimensional visualizations were generated with factoextra and plotly packages, respectively.

### 2.2. Differential Expression Analysis

After merging the normalized datasets, differentially expressed genes (DEGs) between the gastritis and controls were identified using the limma package based on an adjusted *p* value < 0.05 and |log_2_ fold change (log_2_ FC)| ≥ 1. Volcano plots of DEGs were constructed using the ggplot2 package. To further illustrate the expression patterns of DEGs across samples, hierarchical clustering and a heatmap were conducted using the pheatmap package.

### 2.3. Weighted Gene Co‐Expression Network Analysis (WGCNA)

WGCNA was conducted via the WGCNA R package to identify co‐expression modules. Detailed procedures are provided in the Supporting Information 1: Methods.

### 2.4. Identification of Mast Cell‐Related DEGs and Construction of Protein–Protein Interaction (PPI) Network

Mast cell‐related genes were integrated with DEGs and WGCNA‐derived candidate genes to identify mast cell‐associated DEGs. PPI analysis was performed to explore potential functional interactions among these genes, and network topology was used to prioritize important regulators. The detailed procedures for gene selection, network construction, and hub gene identification are described in the Supporting Information 1: Methods.

### 2.5. Functional Enrichment Analysis

To explore the biological functions and signaling pathways associated with the mast cell‐related genes, Gene Ontology (GO) and Kyoto Encyclopedia of Genes and Genomes (KEGG) enrichment analyses were conducted using the clusterProfiler R package. Gene annotations from the org.Mm.eg.db database served as the reference background. GO enrichment was considered significant under the conditions of gene set size ranging from 5 to 5000, a *p*‐value below 0.05, and a false discovery rate (FDR) less than 0.25. GO terms were categorized into biological process (BP), cellular component (CC), and molecular function (MF). For KEGG pathway analysis, the most up‐to‐date gene annotations were obtained using the KEGG Representational State Transfer Application Programming Interface (REST API) (https://www.kegg.jp/kegg/rest/keggapi.html). Significantly enriched pathways were assessed based on *p*‐value, gene count, and gene ratio.

### 2.6. Identification of Hub Genes Using Machine Learning Algorithms

To screen hub genes associated with gastritis, three machine learning algorithms were applied, including least absolute shrinkage and selection operator (LASSO), support vector machine‐recursive feature elimination (SVM‐RFE), and Boruta. A Venn diagram was generated to visualize the overlapping gene sets identified through LASSO, SVM‐RFE, and Boruta algorithms. Detailed information regarding model construction is provided in the Supporting Information 1: Methods.

### 2.7. Receiver Operating Characteristic (ROC) Curve Analysis

To assess the diagnostic performance of hub genes, we performed ROC curve analysis based on the merged expression matrix using the pROC package. An area under the curve (AUC) value greater than 0.7 was considered indicative of good diagnostic potential.

### 2.8. Culture of HP

The HP strain HPSS1, obtained from the American Type Culture Collection (ATCC, Manassas, VA, USA), was cultured on Columbia blood agar plates supplemented with sterile defibrinated sheep blood and HP selective supplement (antibiotic mixture). Bacterial cultures were incubated at 37°C for 48–72 h under microaerophilic conditions (5% O_2_, 10% CO_2_, and 85% N_2_) using a gas‐controlled incubator.

### 2.9. Experimental Animals

6‐week‐old male C57BL/6 mice (18–20 g), purchased from SPF Biotechnology Co., Ltd. (Beijing, China), were housed under specific pathogen‐free conditions with a 12 h light/dark cycle and ad libitum access to food and water. All experimental protocols were performed in accordance with the National Institutes of Health Guide for the Care and Use of Laboratory Animals and approved by the Institutional Animal Ethics Committee of the General Hospital of Ningxia Medical University (Approval No. KYLL‐2025‐0934). To achieve efficient and sustained suppression of FOS expression in vivo, an adeno‐associated virus (AAV)‐mediated shRNA approach was employed, which is essential for evaluating long‐term inflammatory and immune responses in vivo. Mice were randomly allocated into four groups (*n* ≥ 6 per group): Sham (normal control group), HP (HP‐infected group), HP + AAV‐NC (HP + negative control AAV), and HP + AAV‐FOS (HP + FOS knockdown AAV). Mice in the HP, HP + AAV‐NC, and HP + AAV‐FOS groups were fasted and deprived of water for 12 h before infection and again for 4 h post‐infection. These mice were then administered 0.5 mL of HP SS1 suspension (3 × 10^9^ CFU/mL) by oral gavage every other day for a total of five doses [21]. Mice received an equal volume (0.5 mL) of HP culture medium without bacteria in the Sham group, administered on the same schedule as the infection groups. Following the final gavage, mice were kept in standard environmental conditions and allowed unrestricted access to food and water for an additional 3 months. Following HP infection, mice in the HP + AAV‐NC and HP + AAV‐FOS groups were intravenously injected with 15 μL of negative control or AAV encoding FOS‐targeting knockdown vector (1 × 10^11^ vg) via the tail vein. Both AAV constructs were obtained from GeneChem (Shanghai, China). All mice were deeply anesthetized by inhalation of 2% isoflurane and subsequently euthanized by cervical dislocation 4 weeks after AAV administration. Serum samples and gastric tissues were collected for further analysis.

### 2.10. HE Staining

Gastric tissues were processed for paraffin embedding and sectioned for HE staining to evaluate histopathological changes. Detailed staining procedures are provided in the Supporting Information 1: Methods.

### 2.11. Giemsa Staining

Gastric tissue sections were fixed, air‐dried, and subjected to Giemsa staining. Briefly, 2–3 drops of Giemsa working solution (Solarbio, Beijing, China) were applied to cover the entire section and incubated for 5 min. Giemsa buffer was then introduced and gently mixed with the staining solution by tilting the slide for another 5 min. The slides were washed quickly with distilled water, air‐dried, and mounted with neutral resin. The stained samples were observed utilizing a light microscope (Olympus). The nuclei appeared in varying shades of purple‐red, while the cytoplasm was light red.

### 2.12. Warthin–Starry Silver Staining

Gastric tissues were fixed in 4% paraformaldehyde, routinely dehydrated, and embedded in paraffin. Sections (4 μm) were deparaffinized, rehydrated, and rinsed three times with distilled water for 1 min each. The sections were then incubated in an acidic silver nitrate solution (covered) at 56°C for 1 h. Then, sections were transferred to the Warthin–Starry staining mixture (covered, B1, B2, and B3 in a 3:9:4 ratio) and maintained at 56°C until the tissue turned light yellow‐brown. The slides were then thoroughly rinsed with preheated distilled water (56°C) for 5 min, dehydrated, cleared with xylene, and mounted with neutral resin. Stained HP organisms appeared as distinct dark brown to black curved rods against a yellow‐brown background when visualized through a light microscope (Olympus).

### 2.13. Immunohistochemistry (IHC) Staining

Deparaffinization of 4 μm paraffin‐embedded gastric sections was performed in xylene, followed by rehydration in graded ethanol and antigen retrieval using citrate buffer (pH 6.0) at 95°C for 15 min. Blocking of endogenous peroxidase activity was achieved by treating the sections with 3% hydrogen peroxide for 10 min. Subsequently, the sections were incubated with 5% bovine serum albumin (BSA) for 30 min, and subsequently incubated overnight at 4°C with the primary antibody against tryptase (1:400; ab134932, Abcam, Cambridge, UK) and forkhead box P3 (FOXP3; 1:400; ab215206, Abcam). Following phosphate‐buffered saline (PBS) rinsing, the tissue sections were treated with the secondary antibody (1:200, goat anti‐rabbit IgG; Abcam, ab6721) for 1 h. Color development was performed using 3,3^′^‐diaminobenzidine (DAB) chromogen, followed by hematoxylin counterstaining, dehydration, and mounting with neutral resin. Images were captured under a light microscope (Olympus), and stained‐positive cells were quantified using ImageJ software.

### 2.14. Cell Culture and Treatment

The human gastric epithelial cell line GES‐1, human mast cell line LAD2, and human naïve CD4^+^ T cells were sourced from iCell Bioscience Biotechnology Co., Ltd. (Shanghai, China). GES‐1 cells were maintained in Roswell Park Memorial Institute (RPMI) 1640 medium (Gibco, Grand Island, USA) supplemented with 10% fetal bovine serum (FBS; Gibco), and grown at 37°C with 5% CO_2_. For HP infection, GES‐1 cells were seeded in antibiotic‐free RPMI 1640 medium and incubated overnight to reach 70%–80% confluence. Colonies of HP were collected and subsequently suspended in RPMI 1640 medium (antibiotic‐free) containing 10% FBS. The concentration of bacteria was assessed by recording the optical density at 600 nm (OD_600_) with a spectrophotometer. The cells were exposed to HP at an MOI of 100 and incubated together for 24 h. LAD2 cells were cultured in StemPro‐34 medium (Gibco) enriched with 100 U/mL penicillin, 100 μg/mL streptomycin, 100 ng/mL human recombinant stem cell factor (hrSCF; PeproTech, Rocky Hill, NJ, USA), 50 ng/mL human recombinant interleukin‐6 (hrIL‐6; PeproTech), and 2 mM L‐glutamine (Gibco). Cells were grown at 37°C with 5% CO_2_. Transient small interfering RNA (siRNA)‐mediated knockdown was used in vitro to allow rapid and controllable suppression of FOS expression for short‐term functional and mechanistic analyses in gastric epithelial cells. GES‐1 cells were categorized into four groups, including the Normal group: untreated GES‐1 cells cultured under standard conditions; HP group: cells infected with HP; HP + si‐NC group: HP‐infected cells subjected to transfection with non‐targeting control siRNA; HP + si‐FOS group: HP‐infected cells transfected with FOS‐targeting siRNA. To knock down FOS expression, GES‐1 cells underwent transfection with siRNA (si‐FOS‐1, si‐FOS‐2, or si‐FOS‐3; GenePharma, Shanghai, China) with Lipofectamine 3000 reagent (Thermo Fisher Scientific, Waltham, MA, USA) as per the manufacturer’s guidelines. si‐NC (GenePharma), a non‐targeting sequence, was utilized for negative control comparison.

In addition to whole HP infection, GES‐1 cells were also stimulated with the specific HP virulence factor cytotoxin‐associated gene A (CagA) to evaluate its direct effect on FOS expression in gastric epithelial cells. Recombinant CagA protein was added to the culture medium at a final concentration of 10 μg/mL. GES‐1 cells were exposed to CagA for different durations (1, 2, 4, and 6 h).

### 2.15. Cell Counting Kit‐8 (CCK‐8) Assay

Cell viability was assessed using the CCK‐8 assay according to the manufacturer’s instructions. Absorbance at 450 nm was measured to evaluate cell viability. Detailed experimental procedures are provided in the Supporting Information 1: Methods.

### 2.16. Co‐Culture Experiments

Human naïve CD4^+^ T cells were seeded in 96‐well plates (1 × 10^5^ cells/well) pre‐coated with anti‐cluster of differentiation 3 (CD3) antibody (2.5 µg/mL) and cultured in the presence of anti‐cluster of differentiation 28 (CD28) antibody (5 µg/mL). To induce Treg cell differentiation, cells were treated with 3 ng/mL transforming growth factor‐β1 (TGF‐β1) and 2 ng/mL interleukin‐2 (IL‐2) for 5 days. LAD2 mast cells were first stimulated with conditioned media derived from GES‐1 cells under different treatments (normal, HP, HP + si‐NC, and HP + si‐FOS) for 24 h. The resulting LAD2‐conditioned media were then collected and applied to human naïve CD4^+^ T cells cultured under suboptimal Treg‐polarizing conditions (anti‐CD3/CD28 stimulation with low‐dose IL‐2 and TGF‐β1). This design allowed assessment of whether mast cell‐derived factors associated with FOS knockdown in HP‐infected epithelial cells further modulate Treg differentiation beyond baseline cytokine‐driven polarization.

### 2.17. Transwell Migration Assay

A Transwell migration assay was performed using 24‐well Transwell inserts with 8.0 μm pore polycarbonate membranes (Corning Inc., Corning, NY, USA). LAD2 cells (2 × 10^4^) were introduced into the upper chambers containing 200 μL serum‐free culture medium. The lower chambers were loaded with 600 μL of conditioned media collected from GES‐1 cells that had undergone one of the following treatments: Normal, HP, HP + si‐NC, or HP + si‐FOS. Cells that had migrated to the lower surface were treated with 4% paraformaldehyde (30 min) and then incubated with 0.5% crystal violet (Solarbio) for 30 min. Migrated cells were captured via a light microscope (Olympus) in five randomly selected fields and quantified using ImageJ software.

### 2.18. Flow Cytometric Analysis

After the indicated treatments, GES‐1 cells from each group (Normal, HP, HP + si‐NC, and HP + si‐FOS) were harvested and resuspended at a density of 1 × 10^5^ cells per sample in cold PBS. Cells were centrifuged at 1000×*g* for 5 min at 4°C, and the supernatant was discarded. The collected cells were resuspended in 195 μL of Annexin V binding buffer (Beyotime), and then 5 μL of Annexin V conjugated with fluorescein isothiocyanate (Annexin V‐FITC) and 10 μL of propidium iodide (PI) staining reagent were added. After gentle mixing, the samples were maintained in the dark at 25°C for 15 min. Stained cells were promptly examined with a CytoFLEX flow cytometer (Beckman Coulter, Brea, CA, USA). Quantification of apoptotic cells was performed by applying FlowJo software (Tree Star Inc., Ashland, OR, USA). To address potential spectral overlap between FITC and PE fluorochromes, fluorescence compensation was performed using single‐stained controls for each antibody‐conjugated fluorochrome. Compensation matrices were calculated automatically using CytExpert software and manually verified. Fluorescence minus one (FMO) controls were also included to assist in gating strategy refinement and minimize false positives in the FOXP3^+^ Treg population. The naïve CD4^+^ T cells were incubated together with LAD2 cell‐conditioned medium derived from GES‐1 supernatant stimulation. After 24 h of incubation, cells were harvested and subjected to surface staining using fluorochrome‐conjugated monoclonal antibodies: anti‐CD4 fluorescein isothiocyanate (CD4‐FITC; 1:100, 11‐0049‐42, Thermo Fisher Scientific) and anti‐CD25 allophycocyanin (CD25‐APC; 1:100, 17‐0259‐42, Thermo Fisher Scientific). Fixation and permeabilization were carried out using the Foxp3/Transcription Factor Staining Buffer Set, and intracellular Foxp3 expression was subsequently detected with PE‐conjugated anti‐mouse antibodies (Foxp3‐PE; Thermo Fisher Scientific). Flow cytometry was performed on a CytoFLEX system (Beckman Coulter), and subsequent data analysis was carried out using FlowJo (Tree Star Inc.).

### 2.19. Enzyme‐Linked Immunosorbent Assay (ELISA)

Levels of interleukin‐6 (IL‐6), TNF‐α, and interleukin‐1 beta (IL‐1β) in serum and cell culture supernatants, as well as histamine, CCL2, interleukin‐13 (IL‐13), and SCF in cell culture supernatants were measured using appropriate ELISA kits (Esebio Biotechnology Co., Ltd., Shanghai, China) according to the manufacturer’s protocol. Absorbance at 450 nm was measured using a microplate reader (BioTek Instruments), and the relative levels of each analyte were quantified according to standard curves.

### 2.20. Quantitative Real‐Time Polymerase Chain Reaction (qRT‐PCR)

Total RNA was isolated from gastric tissues and cells, reverse‐transcribed into cDNA, and gene expression was quantified by qRT‐PCR. Primer sequences are listed in Supporting Information 2: Table S1, and detailed procedures are provided in the Supporting Information 1: Methods.

### 2.21. Quantification of the HP glmM Gene Copy Number

Gastric mucosal tissues were harvested from mice in each group. Total genomic DNA was extracted using a DNA extraction kit (Tiangen) according to the manufacturer’s instructions. The copy number of HP was quantified by qPCR targeting the glmM gene, a conserved housekeeping gene of HP. qPCR was performed using SYBR Green Master Mix (Tiangen) on a real‐time PCR system. A 294 bp internal fragment of the glmM gene was amplified using primers listed in Supporting Information 2: Table S2.

### 2.22. Western Blot

Protein expression in gastric tissues and cells was analyzed by Western blotting. Band intensities were quantified using ImageJ software and normalized to GAPDH. Detailed experimental procedures and antibody information are provided in the Supporting Information 1: Methods.

### 2.23. Statistical Analysis

GraphPad Prism software (version 8.0; GraphPad Software Inc., San Diego, CA, USA) was utilized for statistical analyses. Experimental data are represented as the mean ± standard deviation (SD), based on no fewer than three independent replicates. Statistical differences between two groups were assessed via an unpaired Student’s *t*‐test, and one‐way analysis of variance (ANOVA) with Tukey’s post hoc test was used for multi‐group analyses. Results were considered statistically significant when *p*  < 0.05.

## 3. Results

### 3.1. Batch Correction and Dimensionality Reduction Confirm Effective Integration of Multi‐Cohort Datasets

To ensure the reliability and comparability of multi‐cohort transcriptomic data, we integrated and normalized three independent datasets (GSE5081, GSE27411, and GSE233973). The density plots before and after normalization showed that gene expression distributions among datasets were successfully aligned post‐processing (Supporting Information 3: Figure S1A). UMAP was employed to assess sample‐level heterogeneity. Prior to correction, samples from different datasets formed distinct clusters. After integration, the samples exhibited a more homogeneous distribution in the UMAP space (Supporting Information 3: Figure S1B). PCA was subsequently conducted to evaluate the overall expression pattern between the disease and control groups. The two‐dimensional (2D)‐PCA plot revealed partial separation between gastritis and normal control samples, with the first two principal components explaining 23% and 13.1% of the total variance, respectively (Supporting Information 3: Figure S1C). In the three‐dimensional (3D)‐PCA space, the separation between groups became more pronounced, confirming the existence of disease‐associated transcriptional profiles (Supporting Information 3: Figure S1D).

### 3.2. Identification of DEGs Between HP‐Induced Gastritis and Normal Controls

After batch correction and dataset integration, a total of 50 gastric tissue samples were included in the downstream analysis, comprising 27 gastritis samples and 23 normal control samples. Applying the criteria of adjusted *p*‐value < 0.05 and |log2 foldchange (FC)| ≥ 1, a total of 939 DEGs were identified. Of these, 739 genes showed significant upregulation, whereas 200 exhibited downregulation (Figure 1A). A hierarchical clustering heatmap was generated to display the expression profiles of the top 20 upregulated and top 20 downregulated DEGs among all samples (Figure 1B).

Figure 1Identification of mast cell‐related hub genes using multiple machine learning algorithms. (A) Volcano plot of DEGs. The *x*‐axis shows the log_2_ fold change (FC), and the *y*‐axis represents the –log_10_ adjusted *p*‐value. Genes with |log_2_ FC| ≥ 1 and adjusted *p*  < 0.05 were considered significantly differentially expressed and are highlighted in red. (B) Heatmap of the top 20 upregulated and top 20 downregulated DEGs, showing hierarchical clustering of samples based on gene expression levels. Each column represents a sample, and each row a gene. Red and blue indicate high and low expression levels, respectively. (C) Least absolute shrinkage and selection operator (LASSO) regression was performed to reduce dimensionality and select predictive genes. The coefficient paths of each gene are plotted against the log‐transformed regularization parameter (λ). (D) Support vector machine‐recursive feature elimination (SVM‐RFE) was applied to identify optimal variables. The plot shows the cross‐validated root mean square error (RMSE) versus the number of retained variables, with the lowest RMSE indicating the best feature set. (E) The Boruta algorithm was employed to rank the importance of candidate genes. Boxplots show the Z‐score importance of each gene. (F) A Venn diagram was used to illustrate the intersection of key genes identified by LASSO, SVM‐RFE, and Boruta.

**Figure:**
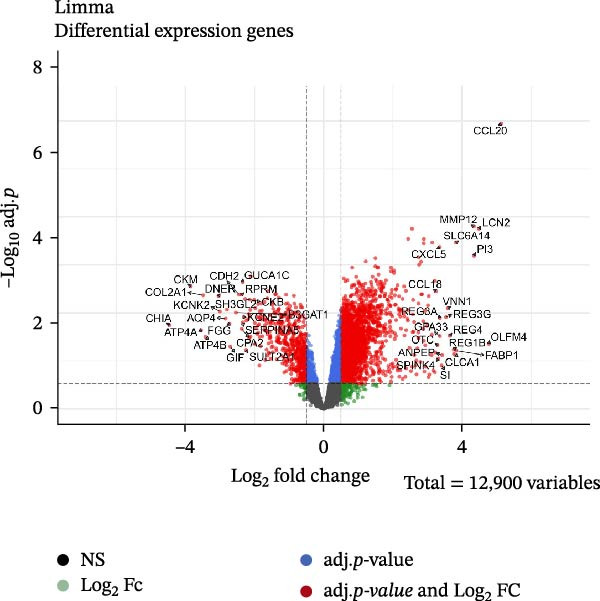
(A)

**Figure:**
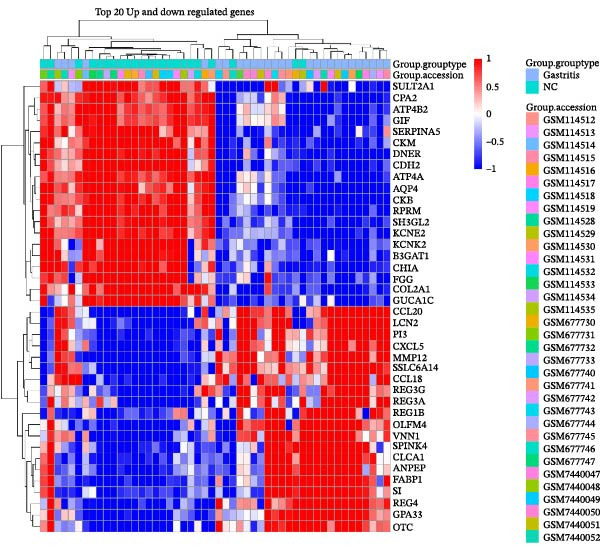
(B)

**Figure:**
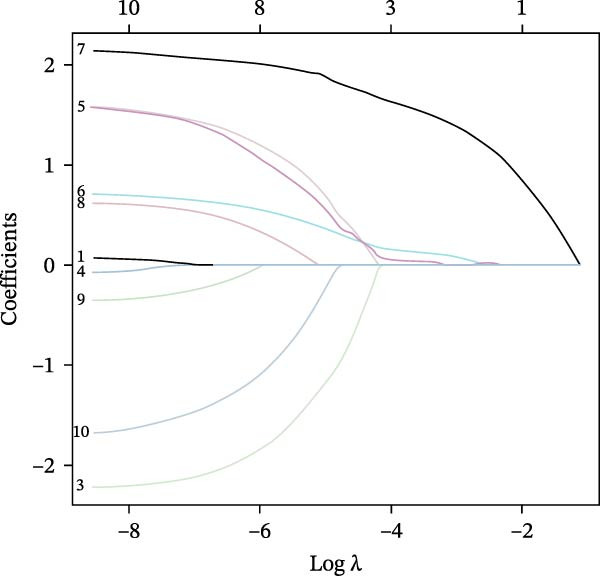
(C)

**Figure:**
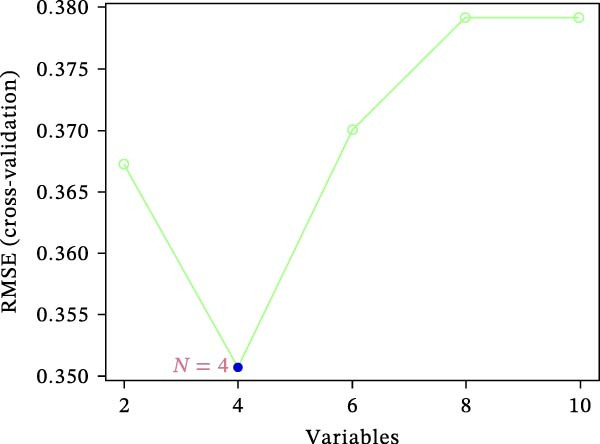
(D)

**Figure:**
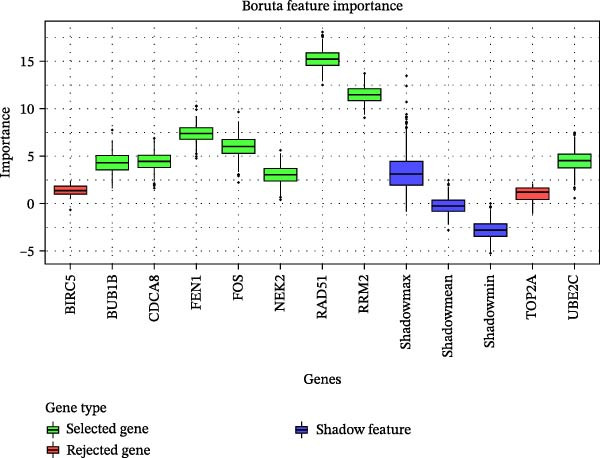
(E)

**Figure:**
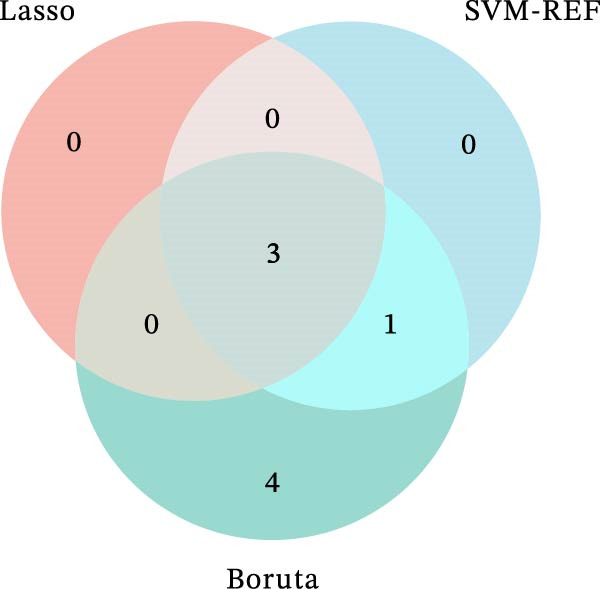
(F)

### 3.3. Hub Co‐Expression Modules Associated With HP‐Induced Gastritis Identified by WGCNA

To identify gene co‐expression networks associated with HP‐induced gastritis, we performed WGCNA. A soft‐thresholding parameter (β) of 8 was selected based on the scale‐free topology criterion (*R* ^2^ = 0.86) and mean connectivity. A minimum module size of 30 genes was used for hierarchical clustering of the gene dendrogram (Supporting Information 3: Figure S2A,B). Gene module detection was carried out through hierarchical clustering and dynamic tree pruning, with the sensitivity parameter set to 3. To reduce redundancy, module eigengenes were computed, and modules with eigengene dissimilarity < 0.25 were merged, resulting in eight co‐expression modules (Supporting Information 3: Figure S2C). Module clustering based on eigengene dissimilarity revealed inter‐module relationships (Supporting Information 3: Figure S2D), and module‐trait correlation analysis identified several modules significantly associated with gastritis status. The purple module showed the highest positive association with the gastritis phenotype (Supporting Information 3: Figure S2E). Using module membership (|MM|) > 0.8 and gene significance (|GS|) > 0.5, 529 highly correlated genes were identified within the purple module. We then evaluated the relationship between MM and GS within the purple module, and a strong positive relationship was observed (Supporting Information 3: Figure S2F).

### 3.4. Identification of Mast Cell‐Related DEGs in HP‐Induced Gastritis

3,345 mast cell‐associated genes were obtained from the GeneCards database. Subsequently, we identified the intersection among mast cell‐related genes, DEGs, and 529 genes from the WGCNA purple module, resulting in 62 overlapping mast‐DEGs (Supporting Information 3: Figure S3A). We then constructed a PPI network of mast‐DEGs using the Search Tool for the Retrieval of Interacting Genes/Proteins (STRING) database. The PPI network was then imported into Cytoscape software for visualization (Supporting Information 3: Figure S3B). Using the degree centrality algorithm, the top 10 hub genes in the PPI network were identified: baculoviral IAP repeat containing 5 (BIRC5), ribonucleotide reductase regulatory subunit M2 (RRM2), DNA topoisomerase II alpha (TOP2A), BUB1 mitotic checkpoint serine/threonine kinase B (BUB1B), RAD51 recombinase (RAD51), ubiquitin conjugating enzyme E2 C (UBE2C), cell division cycle associated 8 (CDCA8), flap structure‐specific endonuclease 1 (FEN1), NIMA related kinase 2 (NEK2), and FOS (Supporting Information 3: Figure S3C).

### 3.5. Functional Enrichment Analysis of 62 Mast‐DEGs

GO and KEGG enrichment analyses were conducted to elucidate the possible biological functions of the 62 mast‐DEGs. In the BP category, enriched terms were predominantly associated with response to stress, response to organic substances, and cell cycle process (Supporting Information 3: Figure S4A). In the CC ontology, mast‐DEGs were mainly localized to the cytosol, extracellular exosome, and collagen‐containing extracellular matrix (Supporting Information 3: Figure S4B). The MF terms were highly involved in anion binding, enzyme binding, and signaling receptor binding (Supporting Information 3: Figure S4C). Furthermore, KEGG pathway analysis revealed that the mast‐DEGs were predominantly enriched in apoptosis, the p53 signaling pathway, and complement and coagulation cascades (Supporting Information 3: Figure S4D).

### 3.6. Identification of Hub Genes Using Machine Learning Algorithms

To further refine the hub mast‐DEGs, three machine learning algorithms were applied to the top 10 candidate genes identified from the PPI network. Through LASSO regression and 10‐fold cross‐validation, three genes, including FOS, RAD51, and RRM2, were identified as the optimal features with non‐zero coefficients (Figure 1C). Subsequently, SVM‐RFE was performed, and based on the model with the minimum root mean square error (RMSE), four genes (FOS, RAD51, RRM2, and FEN1) were selected as key features (Figure 1D). In addition, the Boruta algorithm confirmed eight genes as important features: BUB1B, CDCA8, FEN1, FOS, NEK2, RAD51, RRM2, and UBE2C (Figure 1E). By intersecting the results of the three feature selection algorithms (LASSO, SVM‐RFE, and Boruta), we identified three overlapping hub genes, including FOS, RAD51, and RRM2 (Figure 1F). To assess the diagnostic value of the three hub genes, ROC curve analysis was performed. All three genes exhibited strong discriminatory power between gastritis patients and controls. RAD51 showed the highest diagnostic accuracy (AUC = 0.890), followed by RRM2 (AUC = 0.852) and FOS (AUC = 0.807) (Supporting Information 3: Figure S5A–C).

### 3.7. Validation of Hub Gene Expressions in the HP‐Induced Gastritis Mouse Model

We established an HP‐induced gastritis mouse model. HE staining demonstrated that in contrast to the Sham group, mice in the HP group presented marked gastric mucosal damage, characterized by epithelial erosion, focal necrosis, and significant reduction and atrophy of gastric glands (Figure 2A). Furthermore, Giemsa staining showed the successful colonization of HP, as evidenced by the accumulation of dark blue‐purple bacteria on the gastric epithelial surface in the HP group (Figure 2B), which was absent in the Sham group. Chronic HP infection is known to trigger pronounced inflammatory responses in the gastric mucosa, characterized by elevated levels of pro‐inflammatory cytokines such as IL‐6, TNF‐α, and IL‐1β [22, 23]. To evaluate the extent of systemic inflammation and validate the inflammatory phenotype in our HP‐induced gastritis model, we measured serum cytokine levels. ELISA showed that IL‐6, TNF‐α, and IL‐1β levels were elevated in the HP group relative to the Sham group (Figure 2C). qRT‐PCR indicated that the expression levels of FOS, RRM2, and RAD51 were markedly increased in the HP group relative to the Sham group (Figure 2D).

Figure 2Histological and molecular validation of *Helicobacter pylori* (HP)‐induced gastritis model and mast cell‐related hub gene expression. (A) Hematoxylin and eosin (HE) staining was performed to assess epithelial integrity (Magnification: 200×; Scale bar: 50 μm). (B) Giemsa staining was used to visualize HP colonization (Magnification: 200×; Scale bar: 50 μm). (C) Quantification of inflammatory cytokines interleukin‐6 (IL‐6), tumor necrosis factor‐α (TNF‐α), and interleukin‐1β (IL‐1β) in serum by enzyme‐linked immunosorbent assay (ELISA). (D) Relative mRNA expression levels of Fos proto‐oncogene (FOS), ribonucleotide reductase regulatory subunit M2 (RRM2), and RAD51 recombinase (RAD51), were assessed by quantitative real‐time polymerase chain reaction (qRT‐PCR) in gastric tissues. *N* ≥ 6. All data are presented as mean ± SD.  ^∗∗^ *p*  < 0.01 vs. Sham group.

**Figure:**
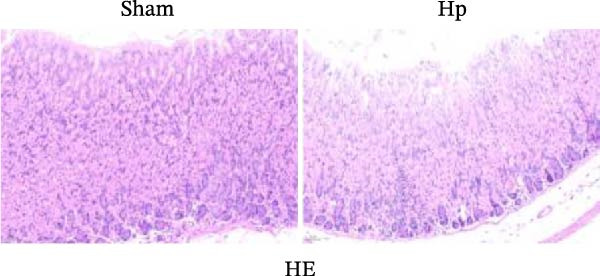
(A)

**Figure:**
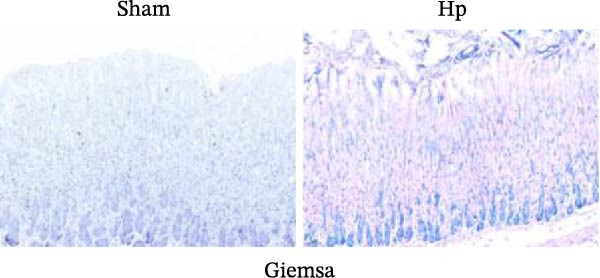
(B)

**Figure:**
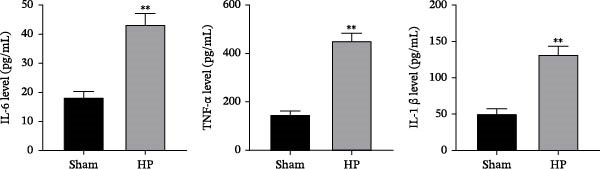
(C)

**Figure:**
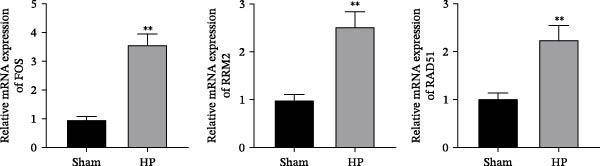
(D)

### 3.8. FOS Knockdown Attenuates Gastric Inflammation, Reduces HP Colonization, and Suppresses Mast Cell and Treg Activation

To investigate the function of FOS in HP‐induced gastritis, mice were infected with HP and received AAV‐delivered shRNA for silencing FOS expression. Analysis via western blot demonstrated that FOS protein expression was elevated in the HP and HP + AAV‐NC groups in comparison with the Sham group, while FOS knockdown via AAV significantly reduced its expression in the HP + AAV‐FOS group (Figure 3A). HE staining revealed that mice in the HP and HP + AAV‐NC groups developed severe gastric injury, characterized by mucosal erosion, focal necrosis, and atrophy of glandular structures. In contrast, the HP + AAV‐FOS group exhibited only mild mucosal shedding and partial recovery of glandular architecture (Figure 3B). Giemsa staining showed a large accumulation of purple‐stained HP on the gastric epithelium in the HP and HP + AAV‐NC groups compared to the Sham group. Bacterial presence was markedly reduced in the HP + AAV‐FOS group compared to the HP + AAV‐NC group (Figure 3C). Consistently, silver staining revealed that, in comparison with the Sham group, greater HP colonization was observed in the gastric tissues in the HP and HP + AAV‐NC groups. Relative to the HP + AAV‐NC group, the number of HP in the gastric mucosa in the HP + AAV‐FOS group was notably diminished (Figure 3D). The glmM gene copy number in the gastric mucosa was significantly increased in HP‐infected mice compared with Sham controls. In contrast, the glmM gene copy number was markedly reduced in the HP + AAV‐FOS group compared with the HP + AAV‐NC group (Figure 3E). ELISA revealed that IL‐6, TNF‐α, and IL‐1β levels in serum were elevated in HP and HP + AAV‐NC mice in comparison to the Sham group. Relative to the HP + AAV‐NC group, IL‐6, IL‐1β, and TNF‐α levels in serum showed a marked decrease in HP + AAV‐FOS mice (Figure 3F). To assess immune cell involvement, IHC was conducted. The expression of Tryptase, a mast cell activation marker, and FOXP3, a Treg differentiation marker, was significantly increased in HP and HP + AAV‐NC gastric tissues compared with the Sham group. Notably, FOS knockdown markedly reduced the abundance of both Tryptase‐ and FOXP3‐positive cells (Figure 3G,H). Together, these results demonstrate that FOS silencing alleviates HP‐induced gastric injury, suppresses inflammatory cytokine release, and inhibits mast cell and Treg cell activation.

Figure 3FOS silencing alleviates HP‐induced gastritis and inflammatory response in mice. (A) Western blot analysis was used to detect FOS protein expression in gastric tissues from four groups: Sham, HP, HP + AAV‐NC (negative control), and HP + AAV‐FOS (FOS knockdown). (B) HE staining was used to evaluate mucosal structure (magnification: 200 ×; Scale bar: 50 μm). (C) Giemsa staining was employed to detect HP colonization (magnification: 200 ×; Scale bar: 50 μm). (D) Warthin‐Starry silver staining was applied to visualize spiral‐shaped HP in gastric mucosal tissues (magnification: 400×; Scale bar: 20 μm). (E) Quantification of HP genomic DNA levels in gastric tissues by qPCR. (F) ELISA of inflammatory cytokines, including IL‐6, TNF‐α, and IL‐1β in serum. (G, H) Immunohistochemical (IHC) staining and semi‐quantification of mast cell tryptase (F) and regulatory T‐cell marker forkhead box P3 (FOXP3) (G) (magnification: 200×; Scale bar: 50 μm). *N* ≥ 6. All data are presented as mean ± SD.  ^∗∗^ *p*  < 0.01 vs. Sham group; ^##^ *p*  < 0.01 vs. HP + AAV‐NC group.

**Figure:**
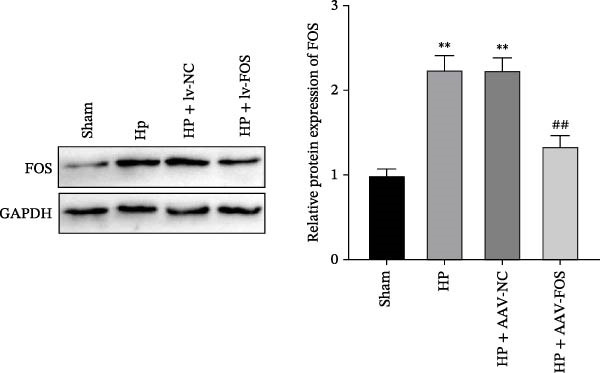
(A)

**Figure:**
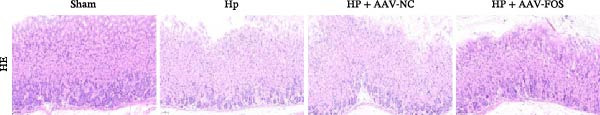
(B)

**Figure:**
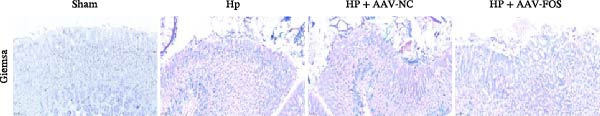
(C)

**Figure:**
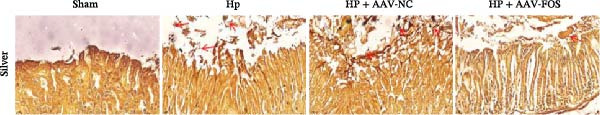
(D)

**Figure:**
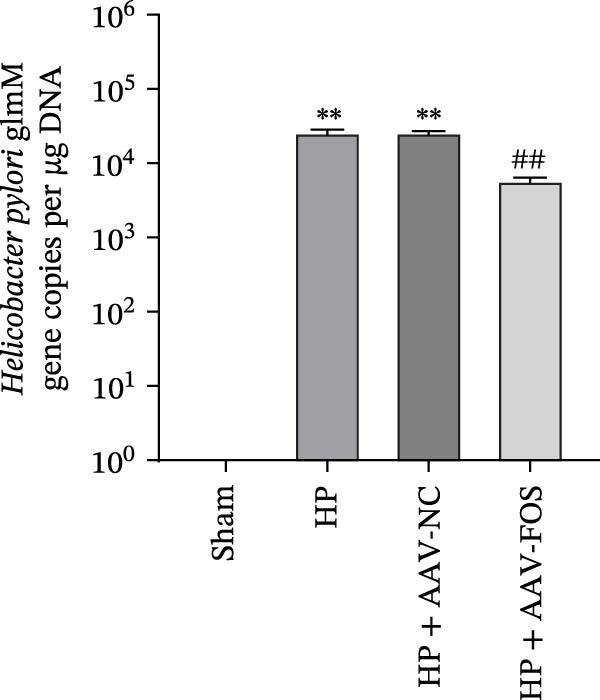
(E)

**Figure:**
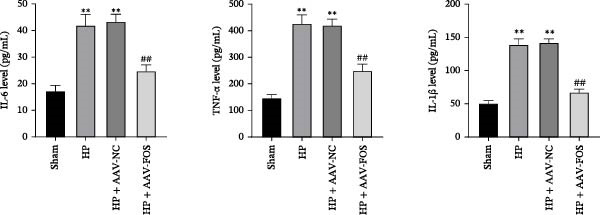
(F)

**Figure:**
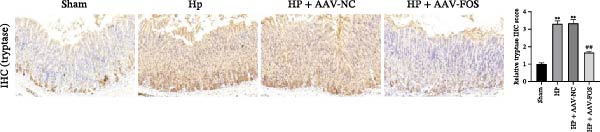
(G)

**Figure:**
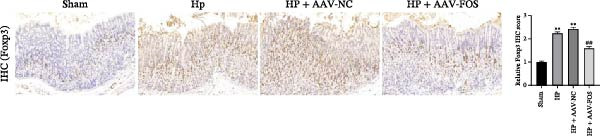
(H)

### 3.9. FOS Knockdown Alleviates Apoptosis and Inhibits Inflammation in HP‐Infected GES‐1 Cells

We then explored the function of FOS in gastric epithelial cells. Treatment with CagA, a major virulence factor of HP, induced a time‐dependent upregulation of FOS protein expression, with a progressive increase observed from 1 to 6 h compared with baseline (0 h) (Figure 4A). siRNA knockdown was employed in HP‐infected human gastric epithelial GES‐1 cells. Western blot analysis revealed that all three siRNAs (si‐FOS‐1, si‐FOS‐2, and si‐FOS‐3) significantly reduced FOS protein levels compared with the si‐NC group, with si‐FOS‐1 exhibiting the highest knockdown efficiency (Figure 4B). Thus, si‐FOS‐1 was selected for further studies. GES‐1 cells were divided into four groups: Normal, HP, HP + si‐NC, and HP + si‐FOS. Western blot analysis demonstrated that FOS expression was higher in the HP and HP + si‐NC groups than in the Normal group. The siRNA‐mediated silencing markedly decreased FOS protein levels in the HP + si‐FOS group in contrast with the HP + si‐NC group (Figure 4C). As opposed to the Normal group, viability of cells was decreased in both the HP and HP + si‐NC groups, while FOS knockdown markedly increased cell viability as compared with the HP + si‐NC group (Figure 4D). Flow cytometry analysis of apoptosis indicated a substantial rise in apoptotic rates in the HP and HP + si‐NC groups in relation to the Normal group. Conversely, a notable decline in apoptosis was observed in the HP + si‐FOS group compared to the HP + si‐NC group (Figure 4E). Western blot analysis revealed elevated expression of BAX and Caspase‐3 in the HP and HP + si‐NC groups in comparison with the Normal group, whereas their expression was notably decreased following FOS knockdown (Figure 4F). In addition to apoptosis, HP infection enhanced the secretion of pro‐inflammatory cytokines (IL‐6, TNF‐α, and IL‐1β), as well as chemokines and mast cell‐related mediators (CCL2, IL‐33, and SCF). Importantly, FOS silencing significantly reduced the levels of these inflammatory mediators compared to the HP + si‐NC group (Figure 4G,H). Collectively, these results indicate that HP/CagA activates FOS expression, and FOS knockdown effectively mitigates HP‐induced cellular injury and inflammatory responses.

Figure 4Knockdown of FOS alleviates HP‐induced apoptosis and inflammation in human gastric epithelial GES‐1 cells. (A) Western blot analysis of FOS protein expression in GES‐1 cells treated with cytotoxin‐associated gene A (CagA) for indicated time points (0, 1, 2, 4, and 6 h). (B) Western blot analysis was applied to the silencing efficiency of FOS in GES‐1 cells transfected with three independent small interfering RNAs (siRNAs; si‐FOS‐1, si‐FOS‐2, and si‐FOS‐3). (C) Western blot analysis of FOS protein expression in four groups of GES‐1 cells: Normal, HP, HP + si‐NC, and HP + si‐FOS. (D) Cell viability was evaluated by the Cell Counting Kit‐8 (CCK‐8) assay across the four groups. (E) Cell apoptosis was determined using the Annexin V‐fluorescein isothiocyanate/propidium iodide (Annexin V‐FITC/PI) dual‐staining method. (F) Western blot detection of pro‐apoptotic proteins BCL2‐associated X protein (BAX) and Cysteine‐aspartic acid protease‐3 (Caspase‐3) in GES‐1 cells of each group. (G, H) ELISA was performed to measure the concentrations of IL‐6, TNF‐α, and IL‐1β, as well as chemokines and inflammation‐associated mediators, including C–C motif chemokine ligand 2 (CCL2), interleukin‐33 (IL‐33), and stem cell factor (SCF), in culture supernatants. All experiments were independently repeated at least three times (*n* ≥ 3). Data are expressed as mean ± SD.  ^∗^ *p*  < 0.05 vs. o h,  ^∗∗^ *p*  < 0.01 vs. Normal group; ^#^ *p*  < 0.05, ^##^ *p*  < 0.01 vs. HP + si‐NC group.

**Figure:**
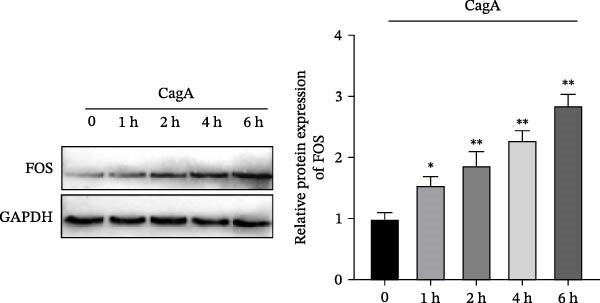
(A)

**Figure:**
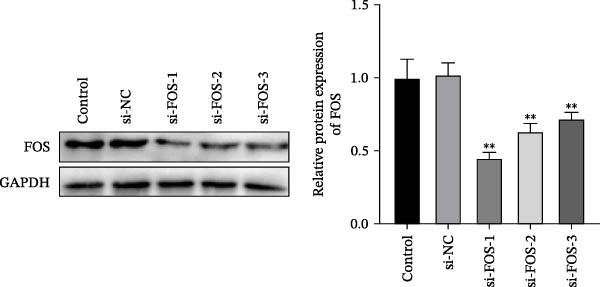
(B)

**Figure:**
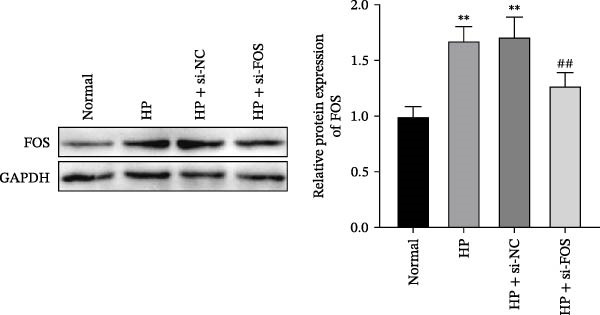
(C)

**Figure:**
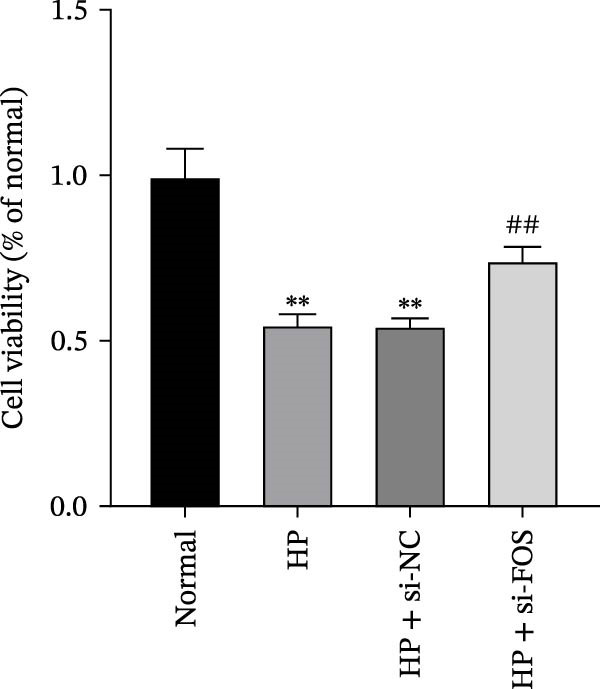
(D)

**Figure:**
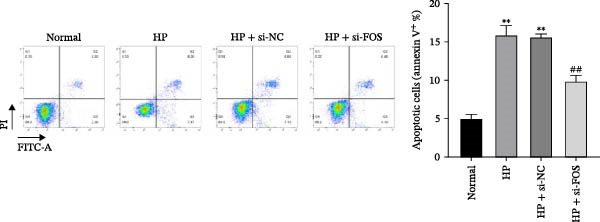
(E)

**Figure:**
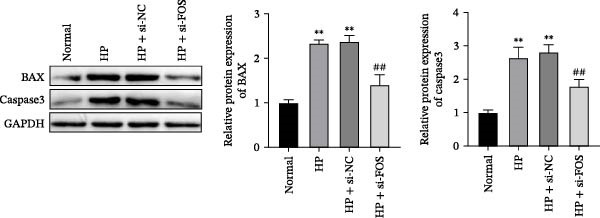
(F)

**Figure:**
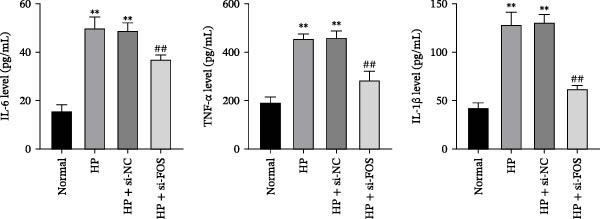
(G)

**Figure:**
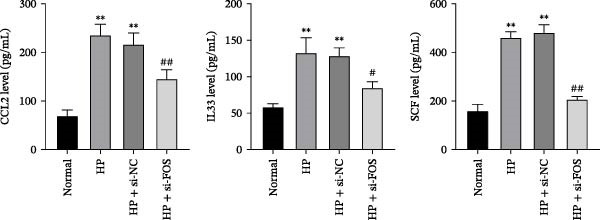
(H)

### 3.10. FOS Knockdown Suppresses Mast Cell Migration and Proinflammatory Mediator Release in Response to HP

Human mast cells (LAD2) were co‐cultured with the supernatants collected from four groups of GES‐1 cells (Normal, HP, HP + si‐NC, and HP + si‐FOS). Transwell migration assays revealed that HP infection promoted mast cell migration in comparison with the Normal group, which was markedly suppressed upon FOS knockdown (Figure 5A). We further examined the secretion of proinflammatory cytokines and chemokines from mast cells under HP infection. The results showed that co‐culture with HP‐conditioned medium increased the levels of CCL2, IL‐6, TNF‐α, and IL‐1β in mast cells compared to the Normal group. Knockdown of FOS significantly suppressed the secretion of CCL2, IL‐6, TNF‐α, and IL‐1β relative to the HP + si‐NC group (Figure 5B). In addition, histamine release, a key marker of mast cell activation, was elevated in response to HP stimulation but declined substantially after FOS knockdown (Figure 5C). Taken together, these findings suggest that silencing FOS may disrupt mast cell‐associated proinflammatory responses in HP‐associated gastritis.

Figure 5FOS knockdown in GES‐1 cells suppresses HP‐induced mast cell migration, activation, and degranulation. (A) Transwell assays were performed to assess the migration of LAD2 mast cells cultured with conditioned media from GES‐1 cells subjected to four treatments: Normal, HP, HP + si‐NC, and HP + si‐FOS. (B) ELISA analysis was conducted to determine mast cell‐related inflammatory mediators, including CCL2, IL‐6, TNF‐α, and IL‐1β, in LAD2 cells after coculture with GES‐1‐derived conditioned media. (C) ELISA quantification of histamine content, a key mast cell degranulation marker, in LAD2 cells across the four groups. A minimum of three independent repetitions was performed for each experiment (*n* ≥ 3). All values are expressed as the mean ± SD.  ^∗∗^ *p*  < 0.01 vs. Normal group; ^##^ *p*  < 0.01 *vs*. HP + si‐NC group.

**Figure:**
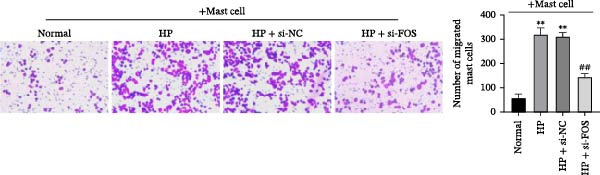
(A)

**Figure:**
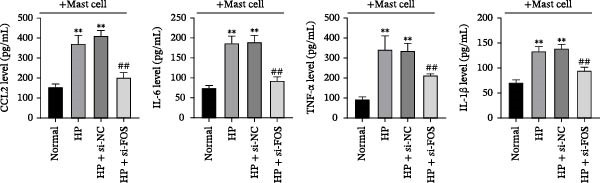
(B)

**Figure:**
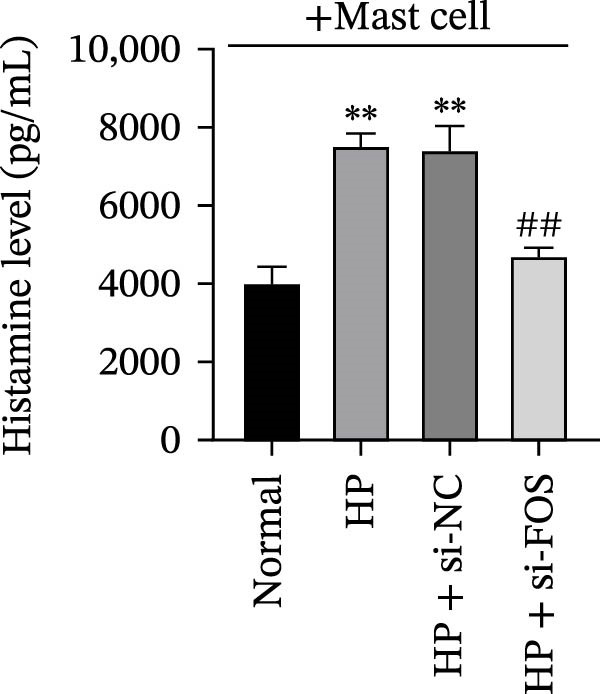
(C)

### 3.11. FOS Knockdown Attenuates Mast Cell Activation‐Associated Treg Cell Differentiation

To assess how GES‐1 cell‐conditioned media influence mast cell‐associated regulation of Treg differentiation, LAD2 cells were co‐cultured with supernatants from four GES‐1 groups (Normal, HP, HP + si‐NC, and HP + si‐FOS). The resulting LAD2 supernatants were then collected and used to culture naïve CD4^+^ T cells under Treg‐inducing conditions, evaluating their effect on Treg differentiation. Flow cytometry analysis revealed that HP infection significantly increased the proportion of FOXP3^+^CD25^+^ Treg cells in CD4^+^ T cell populations compared to the Normal group. However, FOS knockdown markedly suppressed Treg differentiation, confirmed by a significant decrease in the frequency of FOXP3^+^CD25^+^ cells (Figure 6A). ELISA results indicated that TGF‐β, a key cytokine participating in Treg polarization, was markedly upregulated in the HP and HP + si‐NC groups relative to the Normal group. Whereas TGF‐β levels were significantly diminished upon FOS silencing compared to the HP + si‐NC group (Figure 6B). Moreover, western blot analysis demonstrated a similar trend in FOXP3 protein expression, with significant upregulation in the HP‐treated groups and a marked decrease following FOS knockdown (Figure 6C). These findings suggest that FOS knockdown in HP‐infected epithelial cells is associated with reduced mast cell‐associated Treg cell differentiation, accompanied by decreased TGF‐β levels and FOXP3 expression.

Figure 6FOS knockdown attenuates mast cell‐associated regulatory T cell (Treg) differentiation in HP‐infected epithelial cells. (A) Flow cytometry analysis of CD4^+^ T cells cocultured with LAD2 mast cells pretreated with conditioned media from GES‐1 cells (Normal, HP, HP + si‐NC, and HP + si‐FOS). (B) ELISA was performed to detect the secretion level of transforming growth factor‐beta (TGF‐β), a key cytokine promoting Treg differentiation, in the CD4^+^ T cell culture supernatant. (C) Western blot analysis of FOXP3 protein expression in CD4^+^ T cells after coculture with LAD2 cells conditioned by different GES‐1 treatment groups. All experiments were independently repeated at least three times (*n* ≥ 3). Data are expressed as mean ± SD.  ^∗∗^ *p*  < 0.01 vs. Normal group; ^##^ *p*  < 0.01 vs. HP + si‐NC group.

**Figure:**
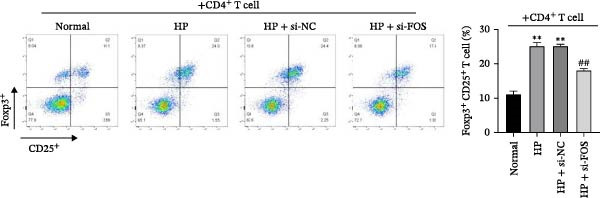
(A)

**Figure:**
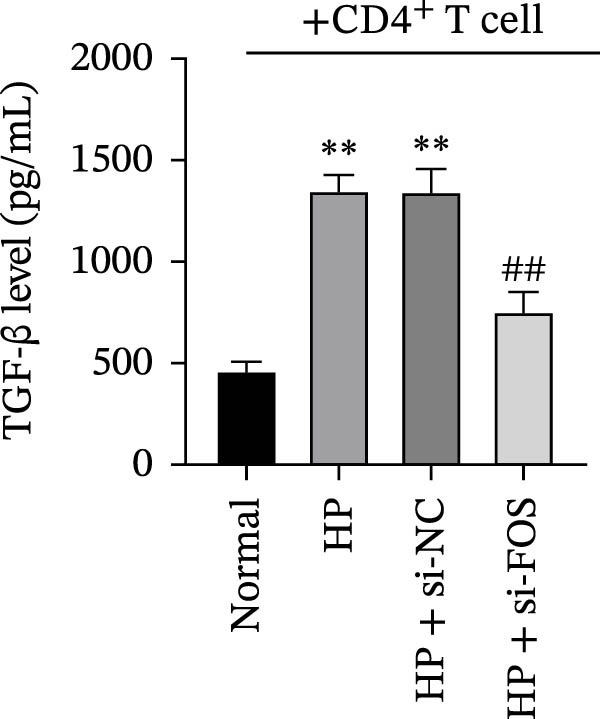
(B)

**Figure:**
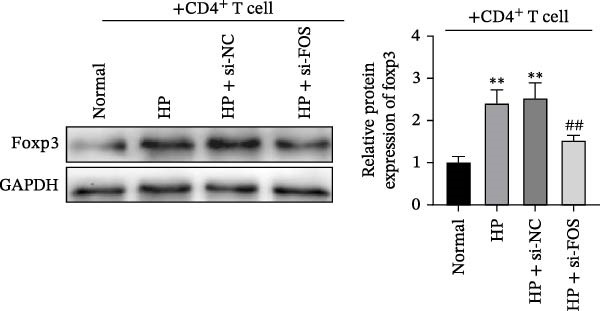
(C)

## 4. Discussion

HP infection remains one of the most prevalent chronic bacterial infections globally and is strongly linked to the pathogenesis of gastritis and gastric malignancies [24]. Beyond direct epithelial injury, growing attention has been directed toward the function of immune dysregulation in driving persistent inflammation [25]. In particular, mast cells and Treg cells have emerged as critical contributors to the immunopathological milieu during HP infection [26, 27], yet the upstream molecular regulators coordinating epithelial–immune cell interactions remain largely undefined. In the present study, we demonstrated that FOS knockdown alleviated gastric mucosal injury and inflammation and effectively suppressed mast cell recruitment and epithelial cell apoptosis. In addition, silencing of the FOS impaired mast cell‐driven Treg induction.

This study identified FOS, RRM2, and RAD51 as mast cell‐associated hub DEGs in gastritis, and these DEGs were significantly upregulated in HP‐induced mouse models. The ribonucleotide reductase small subunit RRM2 is a key enzyme in nucleotide metabolism that converts ribonucleotides to deoxynucleotides, maintaining balanced deoxyribonucleotide triphosphate (dNTP) pools [28]. A recent study has indicated that HP downregulates RRM2 expression, causing dNTP depletion and replication stress‐induced DNA damage, largely independent of CagA [29]. However, our findings revealed an increase in RRM2 levels in HP‐infected gastric tissues, which may reflect a compensatory response. In addition, RRM2 expression is associated with the infiltration of various immune cells and the expression of immune checkpoint molecules [27]. These findings suggest that RRM2 may be indispensable for preserving genomic integrity under HP‐induced stress and modulating the gastric immune microenvironment. RAD51, an ATP‐dependent recombinase, is essential for the early recognition and processing of DNA double‐strand breaks during homologous recombination (HR) repair [30]. Infection with CagA‐positive HP suppresses RAD51 expression and impairs the cytoplasmic‐to‐nuclear translocation of breast cancer susceptibility protein 1 (BRCA1), leading to replication fork instability and HR defects [31]. During HP infection, inhibition of autophagy leads to excessive DNA damage signaling and gastric carcinogenesis progression through RAD51 ubiquitination‐dependent mechanisms [32]. In immunology, RAD51 expression shows a positive correlation with HR deficiency, tumor mutational burden, and neoantigen load, all of which are associated with increased immune cell infiltration [33]. Based on these data, HP infection may induce compensatory upregulation of DNA repair‐related genes, which sustain genomic maintenance and reshape the immune landscape of the gastric mucosa [29, 34]. Although RRM2 and RAD51 were identified as mast cell‐associated hub genes, the present study primarily focused on elucidating epithelial‐immune crosstalk during HP infection. Given that FOS is a stress‐responsive transcription factor with established roles in inflammatory gene regulation and immune cell activation, it was selected for further functional investigation. In contrast, RRM2 and RAD51 are predominantly involved in cell cycle regulation and DNA damage repair, and their potential contributions to immune modulation in HP‐induced gastritis warrant dedicated investigation in future studies. It is reported that c‐Fos is markedly overexpressed in response to HP infection at an early stage, with its upregulation detectable in precancerous [35]. ROC analysis further confirmed its diagnostic potential in this study, with FOS yielding an AUC value of 0.807. Accordingly, analysis of FOS gene or c‐Fos protein expression in biopsy specimens could provide a valuable indicator of gastritis activity and malignant transformation risk. In CAG, the expression level of c‐FOS is significantly elevated, and Periostracum cicadae can inhibit epithelial–mesenchymal transition by blocking the c‐Fos/c‐Jun signaling pathway [36]. Aligned with these findings, we revealed that FOS expression was significantly elevated in both HP‐infected gastric epithelial cells and gastric tissues. As the first line of defense, the gastric epithelium forms a physical and immunological barrier between the host and luminal microbes. HP establishes persistent colonization by adhering to gastric epithelial cells via specific host receptors [37]. Consistent with this, our study demonstrated that HP infection impaired epithelial integrity, while FOS silencing enhanced cell viability, suppressed apoptosis in HP‐infected epithelial cells. Furthermore, FOS knockdown was associated with attenuated mucosal inflammation, reduced bacterial burden, and decreased mast cell activation and Treg‐associated responses. Therefore, FOS may serve as a diagnostic biomarker and an immune regulator in HP‐induced gastritis.

Upon stimulation by exogenous or endogenous antigens, innate immune cells, such as macrophages, mast cells, dendritic cells, and innate lymphoid cells, are activated and recruited to the gastric mucosa, initiating inflammatory and immune responses [38]. If this immune regulation becomes dysregulated, it can lead to chronic inflammation and fibrosis, thereby promoting aberrant proliferation of mucosa‐associated lymphoid tissue, a phenomenon commonly observed in chronic HP infection [38]. HP infection and metabolic syndrome have been implicated in mast cell activation, which triggers inflammatory signaling pathways that not only contribute to neurodegenerative processes but are also associated with adverse pregnancy and neonatal outcomes [39, 40]. Moreover, IL‐33 secreted by gastric epithelial cells enhances TNF‐α release from mast cells, and the resulting increase in TNF‐α impairs epithelial regeneration, contributing to the advancement of HP‐induced gastritis and persistent colonization [41]. Additionally, it is reported that SCF can sustain mast cell recruitment, survival, and activation through c‐Kit signaling [42]. In our study, HP infection increased mast cell migration and the release of proinflammatory mediators, including CCL2, IL‐6, TNF‐α, and IL‐1β, accompanied by elevated histamine release. This was accompanied by elevated levels of IL‐33 and SCF. Conversely, FOS knockdown significantly suppressed these mast cell‐associated inflammatory responses. Together, these findings indicate that epithelial FOS expression is closely associated with the production of mast cell‐activating mediators and the amplification of mast cell‐driven inflammation during HP infection. Furthermore, through modulation of mast cell activity, the desensitization process facilitates enhanced induction and proliferation of Treg cells [43]. Elevated levels of Treg cells are linked to mast cell accumulation in eosinophilic esophagitis [44]. Angiopoietin‐like 4 (ANGPTL4) promotes Treg cell proliferation through interaction with integrin subunit alpha V (ITGAV), exacerbating HP‐induced gastritis [45]. Furthermore, HP enhances IL‐6 production in dendritic cells and macrophages via activation of nuclear factor kappa B (NF‐κB) signaling, which results in engagement of interleukin‐17A (IL‐17A) expression in FOXP3^+^ T cells. In addition, the abundance of CD4^+^IL‐17A^+^FOXP3^+^ T cells correlated positively with advanced gastric precancerous lesions [46]. Our study demonstrated that HP infection promoted Treg polarization, as evidenced by the elevated proportion of CD25^+^FOXP3^+^ T cells and increased secretion of TGF‐β. Importantly, FOS knockdown in HP‐infected epithelial cells markedly reduced mast cell‐associated Treg cell differentiation, along with decreased TGF‐β levels and FOXP3 expression. Collectively, these findings identify FOS as a novel epithelial‐derived regulator of mast cell‐associated Treg polarization in HP‐induced gastritis, highlighting its potential as a therapeutic target for controlling chronic HP infection and associated gastric inflammation.

As a proto‐oncogene product, FOS has been widely studied in gastric carcinogenesis [47, 48]; however, its role in HP‐induced inflammation and immune regulation has rarely been reported. Our study showed that silencing of the FOS gene attenuated HP‐induced gastritis by suppressing mast cell activation and Treg cell polarization. This finding links c‐Fos to two key immune cell types involved in HP infection: mast and Treg cells. Notably, a range of monoclonal antibodies and small‐molecule inhibitors have been developed to target mast cells or their downstream mediators specifically, such as mas‐related G‐protein coupled receptor X2 (MRGPRX2) antagonists and tyrosine kinase inhibitors, which are currently undergoing clinical trials [45]. T‐5224, a c‐Fos/AP‐1 inhibitor, mitigates IgE‐induced mast cell activation and allergic inflammation [18]. Together with our findings, these studies indicate that pharmacological inhibition of FOS or its downstream signaling may represent a promising approach to mitigate HP‐associated chronic gastritis.

Several limitations of the present study should be acknowledged. First, although mast cell activation and Treg polarization were examined, other immune components involved in HP‐associated gastritis were not systematically evaluated. Second, bacterial colonization was assessed using histological analysis and relative burden measurements rather than longitudinal eradication or clearance assays. Third, while alterations in TGF‐β levels and FOXP3 expression were observed, the precise cellular sources of TGF‐β and the signaling pathways governing Treg differentiation were not directly dissected. Future studies are therefore warranted to establish causal mechanisms and to integrate these findings within a broader immune regulatory framework.

## 5. Conclusion

By integrating multi‐cohort transcriptomic datasets and applying WGCNA and machine learning analyses, we identified FOS, RRM2, and RAD51 as mast cell‐related hub genes in HP‐induced gastritis. Among them, FOS emerged as the pivotal regulatory gene linking epithelial injury, inflammatory responses, and immune modulation. Targeting FOS may provide a novel therapeutic approach for the prevention and treatment of HP‐associated gastritis.

## Author Contributions

**Wen Ma:** conceptualization; formal analysis; methodology; writing – original draft; validation; resources; **Ruidong Han and Lei Wang:** formal analysis; methodology; validation; editing.

## Funding

This study was supported by Key R&D Program Project of Ningxia Hui Autonomous Region; Project Name: Establishment and Application of a New Individualized Diagnosis and Treatment System for PDO and CTC in Gastric Cancer Organoids; Project Number: 2021BEG03037.

## Disclosure

All authors have read and approved the manuscript.

## Ethics Statement

The experiments conformed to the Guide for the Care and Use of Laboratory Animals. Animal study has been approved by the Animal Ethics Committee of General Hospital Of Ningxia Medical University (No. KYLL‐2025‐0934). All methods are reported in accordance with ARRIVE guidelines.

## Consent

The authors have nothing to report.

## Conflicts of Interest

The authors declare no conflicts of interest.

## Supporting Information

Additional supporting information can be found online in the Supporting Information section.

## Supporting information


**Supporting Information 1** Materials and Methods.

**Supporting Information 2** Supporting Table 1 Primer sequences used in this study. Supporting Table 2 Primer sequences for *Helicobacter pylori* glmM gene amplification.

**Supporting Information 3** Supporting Figure 1. Data preprocessing and dimensionality reduction of merged Gene Expression Omnibus (GEO) datasets. (A) Density plots before (left) and after (right) normalization across three GEO datasets (GSE5081, GSE27411, and GSE233973), showing the distribution of gene expression values. (B) Uniform Manifold Approximation and Projection (UMAP) analysis of the three normalized datasets. (C) Principal component analysis (PCA) of gastritis and control samples, visualized with 95% confidence ellipses. (D) Three‐dimensional PCA plot showing the separation between the gastritis and normal control (NC) groups based on PC1, PC2, and PC3. Supporting Figure 2. Weighted gene co‐expression network analysis (WGCNA) for module identification and correlation. (A‐B) Determination of the soft‐thresholding power. Scale‐free topology fit index (A) and mean connectivity (B) were plotted to select an appropriate soft‐threshold. (C) Dendrogram of genes clustered based on a topological overlap matrix (TOM), with corresponding module colors assigned using dynamic tree cut and subsequent merging. (D) A dendrogram of module eigengenes and an adjacency heatmap were constructed to illustrate inter‐module similarity. (E) Module–trait relationship analysis illustrating correlations between module eigengenes and clinical traits. Correlation coefficients are shown within each cell, with color intensity reflecting the strength and direction of the association. (F) Scatter plot of gene significance versus module membership in the selected key module. Supporting Figure 3. Identification and interaction analysis of mast cell‐related DEGs. (A) Venn diagram showing the intersection among DEGs, WGCNA‐derived hub module genes, and mast cell‐related genes obtained from the GeneCards database. (B) Protein–protein interaction (PPI) network of the intersected mast cell‐related DEGs constructed using the STRING database and visualized in Cytoscape. (C) Identification of hub genes within the PPI network using the degree algorithm in Cytoscape. The color intensity represents the ranking score of each gene node. Supporting Figure 4. Functional enrichment analysis of mast cell‐related DEGs. (A–C) Gene Ontology (GO) enrichment in the biological process (BP) category, cellular component (CC) category, and molecular function (MF) category. (D) Kyoto Encyclopedia of Genes and Genomes (KEGG) pathway enrichment analysis of mast cell‐related DEGs. Bubble size indicates gene count, and the color gradient represents the statistical significance level expressed as the negative logarithm (base 10) of the false discovery rate (–log10 FDR). Supporting Figure 5. Diagnostic performance of three mast cell‐related hub genes based on receiver operating characteristic (ROC) analysis. (A–C) ROC curves were plotted to evaluate the diagnostic accuracy of FOS, RAD51, and RRM2, respectively, using the combined expression matrix from merged datasets. The area under the curve (AUC) was calculated to quantify the sensitivity and specificity of each gene in distinguishing disease from control samples.

## Data Availability

The datasets used and/or analyzed during the current study are available from the corresponding author upon reasonable request.
