# Supplementary material for: FOS Knockdown Alleviates Helicobacter pylori‐Infected Gastritis by Suppressing Mast Cell Activation and Treg Polarization
Source: Mediators Inflamm. 2026 Mar 23;2026:4596288. doi: 10.1155/mi/4596288 (PMC13140227; doi:10.1155/mi/4596288)
Supplement: Supplementary file 1 — Supporting Information 1 Materials and Methods. [file MI-2026-4596288-s003.docx]

**Weighted gene co-expression network analysis (WGCNA)**

WGCNA was conducted via the WGCNA R package to identify co-expression modules. The median absolute deviation (MAD) was computed for each gene, and the bottom 50% of genes with the lowest variance were removed. Outlier samples and genes were filtered with the goodSamplesGenes function. A soft-thresholding power (β) was selected based on the scale-free topology criterion. An adjacency matrix was constructed and transformed into a topological overlap matrix (TOM), which was used to calculate dissimilarity and perform average linkage hierarchical clustering. The dynamic tree cut algorithm was utilized to delineate modules, with a minimum cluster size of 30. Modules exhibiting strong eigengene correlations (dissimilarity < 0.25) were combined. Module membership (MM) and gene significance (GS) were calculated for each gene to evaluate intramodular connectivity and their association with clinical traits, respectively. Genes meeting the thresholds of |MM| > 0.8 and |GS| > 0.5 in the important module were identified as candidate genes for further analysis.

**Identification of mast cell-related DEGs and construction of protein-protein interaction (PPI) network**

Mast cell-related genes were collected from the GeneCards database (https://www.genecards.org/), and those with relevance scores above the average were selected to form the mast cell gene set. By intersecting this gene set with the DEGs and the candidate genes identified from WGCNA, mast cell-specific DEGs were obtained. To examine the potential interactions among these genes, a PPI network was established through the Search Tool for the Retrieval of Interacting Genes/Proteins (STRING) database (https://string-db.org/) with a minimum confidence score threshold of 0.4. The resulting network was visualized using Cytoscape software (version 3.9.1; https://cytoscape.org/). The CytoHubba plugin for Cytoscape was then applied to rank genes based on the degree centrality algorithm, and the top 10 genes were identified as potentially important regulators.

**Identification of hub genes using machine learning algorithms**

To screen hub genes associated with gastritis, three machine learning algorithms were applied, including least absolute shrinkage and selection operator (LASSO), support vector machine-recursive feature elimination (SVM-RFE), and Boruta. LASSO regression was conducted using the glmnet R package to select genes with the highest predictive power. The optimal regularization coefficient (λ) was selected through 10-fold cross-validation. The SVM-RFE algorithm, executed using the e1071 package in R, was applied to iteratively discard non-contributory features. The subset of genes associated with the model exhibiting the lowest root mean square error (RMSE) in 10-fold cross-validation was identified as the optimal feature set. Feature selection was further refined using the Boruta algorithm implemented in the Boruta R package, which applies a random forest-based classification framework. A Venn diagram was generated to visualize the overlapping gene sets identified through LASSO, SVM-RFE, and Boruta algorithms.

**Hematoxylin and eosin (HE) staining**

Gastric tissues were collected and subjected to fixation in 4% paraformaldehyde for 24 h, followed by dehydration, paraffin embedding, and microtome sectioning at 4 μm thickness. Sections underwent hematoxylin staining (Beyotime, Shanghai, China) for 5 min, acid alcohol differentiation, and eosin counterstaining for 2 min. Following dehydration, clearing, and mounting with neutral resin, histological alterations were examined with a light microscope (Olympus, Tokyo, Japan).

**Cell Counting Kit-8 (CCK-8) assay**

Different treatment groups of GES-1 cells were plated in 96-well plates at a concentration of 5 × 10³ cells/well and maintained at 37°C for 24 h. Subsequently, 90 μL of fresh medium mixed with 10 μL of CCK-8 reagent (Beyotime) was added to replace the original medium, and then incubated for 2 h at 37°C. Measurement of absorbance at 450 nm was carried out using a microplate reader (BioTek Instruments, Winooski, VT, USA).

**Quantitative real-time polymerase chain reaction (qRT-PCR)**

RNA extraction was carried out from gastric tissues and cells using TRIzol (Thermo Fisher Scientific), and cDNA was synthesized using the FastKing RT Kit (Tiangen, Beijing, China). qPCR assays were conducted with SuperReal PreMix Plus (SYBR Green) (Tiangen) on a 7500 system (Thermo Fisher Scientific). The reaction conditions included an initial heating step at 95°C for 30 sec, followed by 40 cycles of denaturation (95°C, 10 sec) and annealing/extension (60°C, 30 sec). Normalization was performed against glyceraldehyde-3-phosphate dehydrogenase (GAPDH), and target gene expression was evaluated by the 2^⁻ΔΔCt^ method. Primer sequences are detailed in Supplementary Table 1.

**Western blot**

Proteins were isolated from gastric tissues and cells utilizing radioimmunoprecipitation assay buffer containing protease inhibitors, and concentrations were quantified via a bicinchoninic acid (BCA) protein assay kit (Yesen, Shanghai, China). Equal amounts of protein were separated on 10% sodium dodecyl sulfate–polyacrylamide gel electrophoresis gels and transferred to polyvinylidene fluoride membranes. Following incubation with 5% non-fat milk for 1 h at room temperature, the membranes were incubated overnight at 4°C with primary antibodies (1:2000; Abcam, Cambridge, UK), followed by horseradish peroxidase (HRP)-conjugated secondary antibodies (goat anti-rabbit IgG: ab6721 or goat anti-mouse IgG: ab97023; Abcam) for 1 h. Protein bands were detected via enhanced chemiluminescence (ECL) reagent (E266188; Aladdin, Shanghai, China) and captured with a Tanon 5200 imaging system (Tanon, Shanghai, China). Band intensity was quantified using ImageJ software. The following primary antibodies were used: FOS (ab222699), Bcl-2-associated X protein (BAX; ab32503), Caspase-3 (ab32351), FOXP3 (ab20034), and GAPDH (ab181602).
