# Supplementary material for: FOS Knockdown Alleviates Helicobacter pylori‐Infected Gastritis by Suppressing Mast Cell Activation and Treg Polarization
Source: Mediators Inflamm. 2026 Mar 23;2026:4596288. doi: 10.1155/mi/4596288 (PMC13140227; doi:10.1155/mi/4596288)
Supplement: Supplementary file 2 — Supporting Information 2 Supporting Table 1 Primer sequences used in this study. Supporting Table 2 Primer sequences for Helicobacter pylori glmM gene amplification. [file MI-2026-4596288-s001.docx]

Supplementary Table 1 Primer sequences used in this study

| Primer name | Sequence (5'-3') |
| --- | --- |
| GAPDH-F | GAGTCAACGGATTTGGTCGT |
| GAPDH-R | TTGATTTTGGAGGGATCTCG |
| si-NC-F | UUCUCCGAACGUGUCACGUTT |
| si-NC-R | ACGUGACACGUUCGGAGAATT |
| si-FOS-1-F | UUCUUCUUCUGGAGAUAACUG |
| si-FOS-1-R | GUUAUCUCCAGAAGAAGAAGA |
| si-FOS-2-F | UCAACAUGCUACUAACUACCA |
| si-FOS-2-R | GUAGUUAGUAGCAUGUUGAGC |
| si-FOS-3-F | UAGUUAAUGCUAUGAGAAGAC |
| si-FOS-3-R | CUUCUCAUAGCAUUAACUAAU |
| FOS-F | GCGCTGTGTTGCTGTAAACA |
| FOS-R | GGGGGAGGAGAAAGCAAGTC |
| RRM2-F | TGATGCCGGGCCTTACATTT |
| RRM2-R | CTAGTGACCAGCCTGGCAAA |
| RAD51-F | GATACGGTCTCTCTGGCAGC |
| RAD51-R | ATGAGCCTGTGAAGAAGCCC |

Supplementary Table 2 Primer sequences for Helicobacter pylori glmM gene amplification

| Primer Name | Sequence (5'-3') |
| --- | --- |
| glmM-F | AGACACCAGAAAAAGCGGCT |
| glmM-R | CCGCACCATTAGCCGTATCT |
|  |  |
