# Supplementary material for: FOS Knockdown Alleviates Helicobacter pylori‐Infected Gastritis by Suppressing Mast Cell Activation and Treg Polarization
Source: Mediators Inflamm. 2026 Mar 23;2026:4596288. doi: 10.1155/mi/4596288 (PMC13140227; doi:10.1155/mi/4596288)

Supplementary Figure 1. Data preprocessing and dimensionality reduction of merged Gene Expression Omnibus (GEO) datasets. (A) Density plots before (left) and after (right) normalization across three GEO datasets (GSE5081, GSE27411, and GSE233973), showing the distribution of gene expression values. (B) Uniform Manifold Approximation and Projection (UMAP) analysis of the three normalized datasets. (C) Principal component analysis (PCA) of gastritis and control samples, visualized with 95% confidence ellipses. (D) Three-dimensional PCA plot showing the separation between the gastritis and normal control (NC) groups based on PC1, PC2, and PC3.


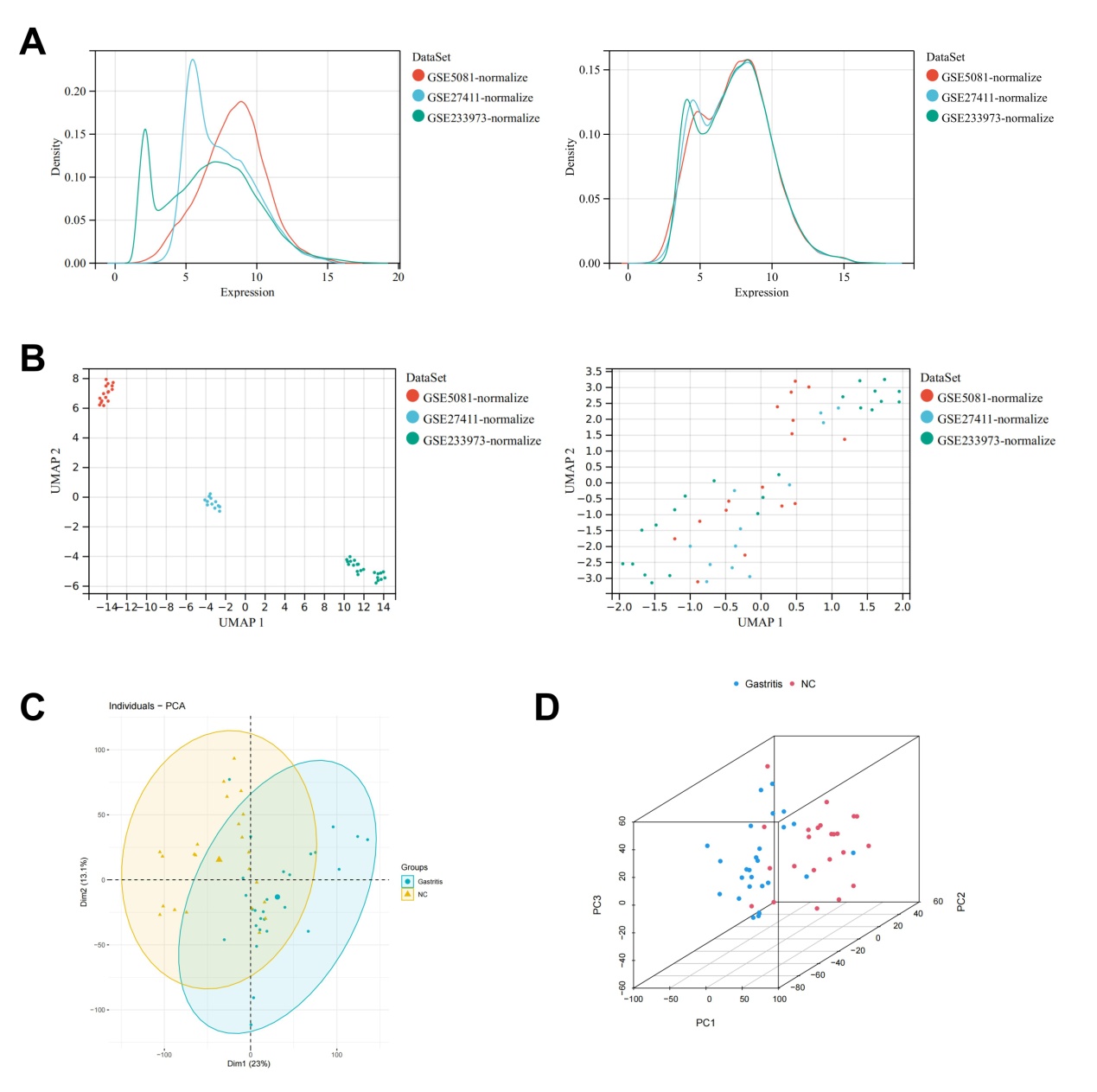


Supplementary Figure 2. Weighted gene co-expression network analysis (WGCNA) for module identification and correlation. (A-B) Determination of the soft-thresholding power. Scale-free topology fit index (A) and mean connectivity (B) were plotted to select an appropriate soft-threshold. (C) Dendrogram of genes clustered based on a topological overlap matrix (TOM), with corresponding module colors assigned using dynamic tree cut and subsequent merging. (D) A dendrogram of module eigengenes and an adjacency heatmap were constructed to illustrate inter-module similarity. (E) Module-trait relationship analysis illustrating correlations between module eigengenes and clinical traits. Correlation coefficients are shown within each cell, with color intensity reflecting the strength and direction of the association. (F) Scatter plot of gene significance versus module membership in the selected key module.


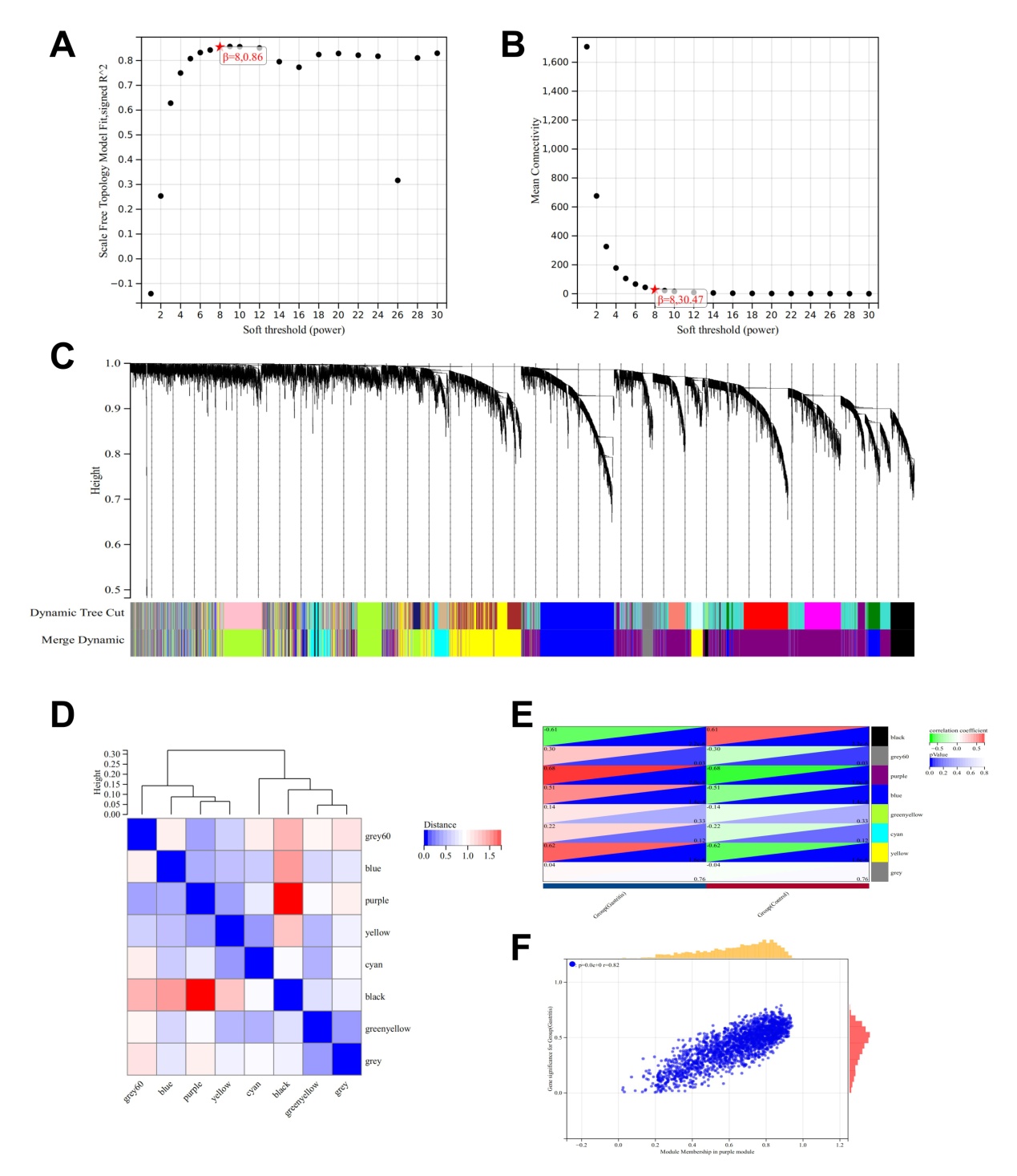


Supplementary Figure 3. Identification and interaction analysis of mast cell-related DEGs. (A) Venn diagram showing the intersection among DEGs, WGCNA-derived hub module genes, and mast cell-related genes obtained from the GeneCards database. (B) Protein-protein interaction (PPI) network of the intersected mast cell-related DEGs constructed using the STRING database and visualized in Cytoscape. (C) Identification of hub genes within the PPI network using the degree algorithm in Cytoscape. The color intensity represents the ranking score of each gene node.


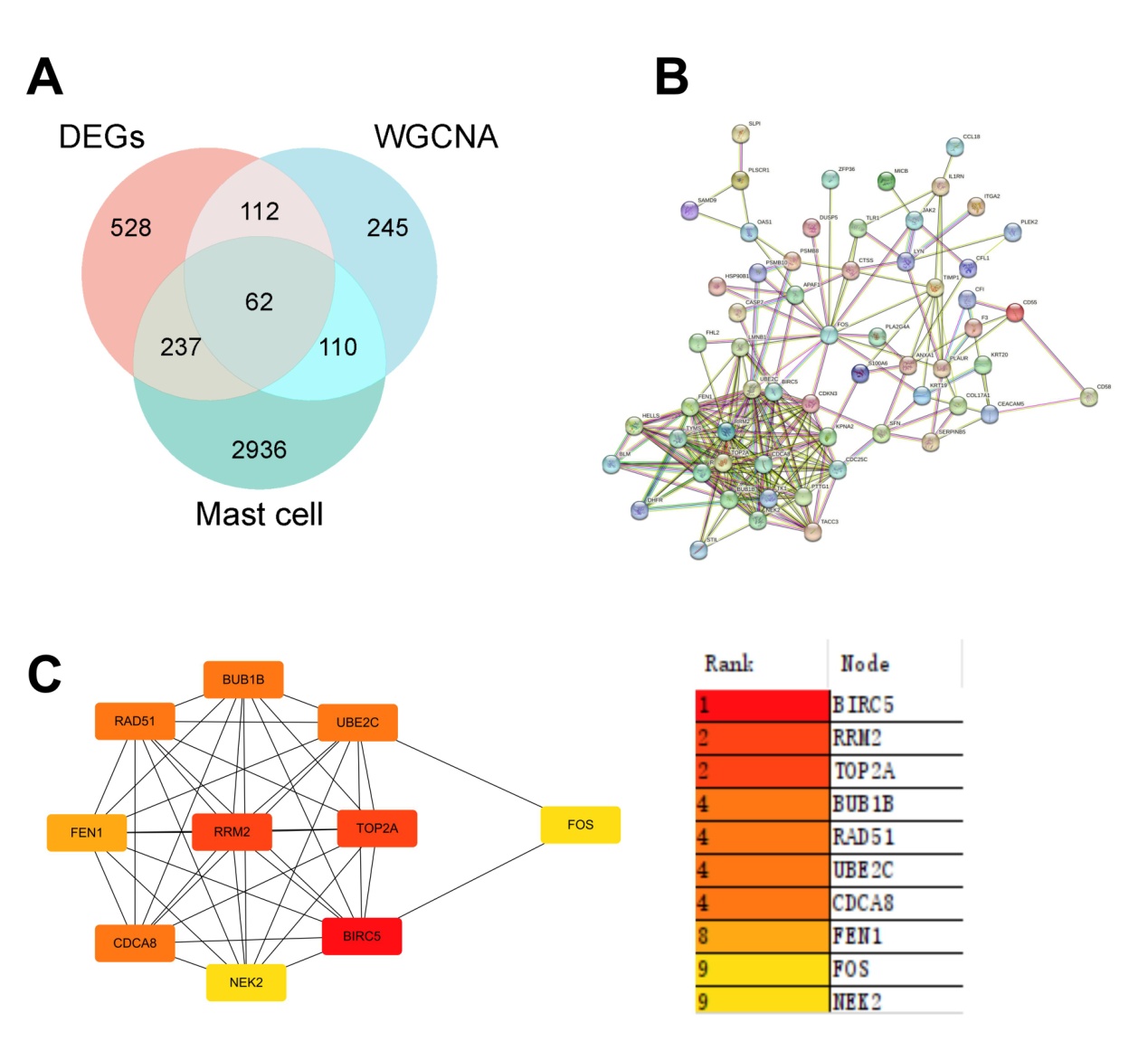


Supplementary Figure 4. Functional enrichment analysis of mast cell-related DEGs. (A-C) Gene Ontology (GO) enrichment in the biological process (BP) category, cellular component (CC) category, and molecular function (MF) category. (D) Kyoto Encyclopedia of Genes and Genomes (KEGG) pathway enrichment analysis of mast cell-related DEGs. Bubble size indicates gene count, and the color gradient represents the statistical significance level expressed as the negative logarithm (base 10) of the false discovery rate (–log₁₀ FDR).


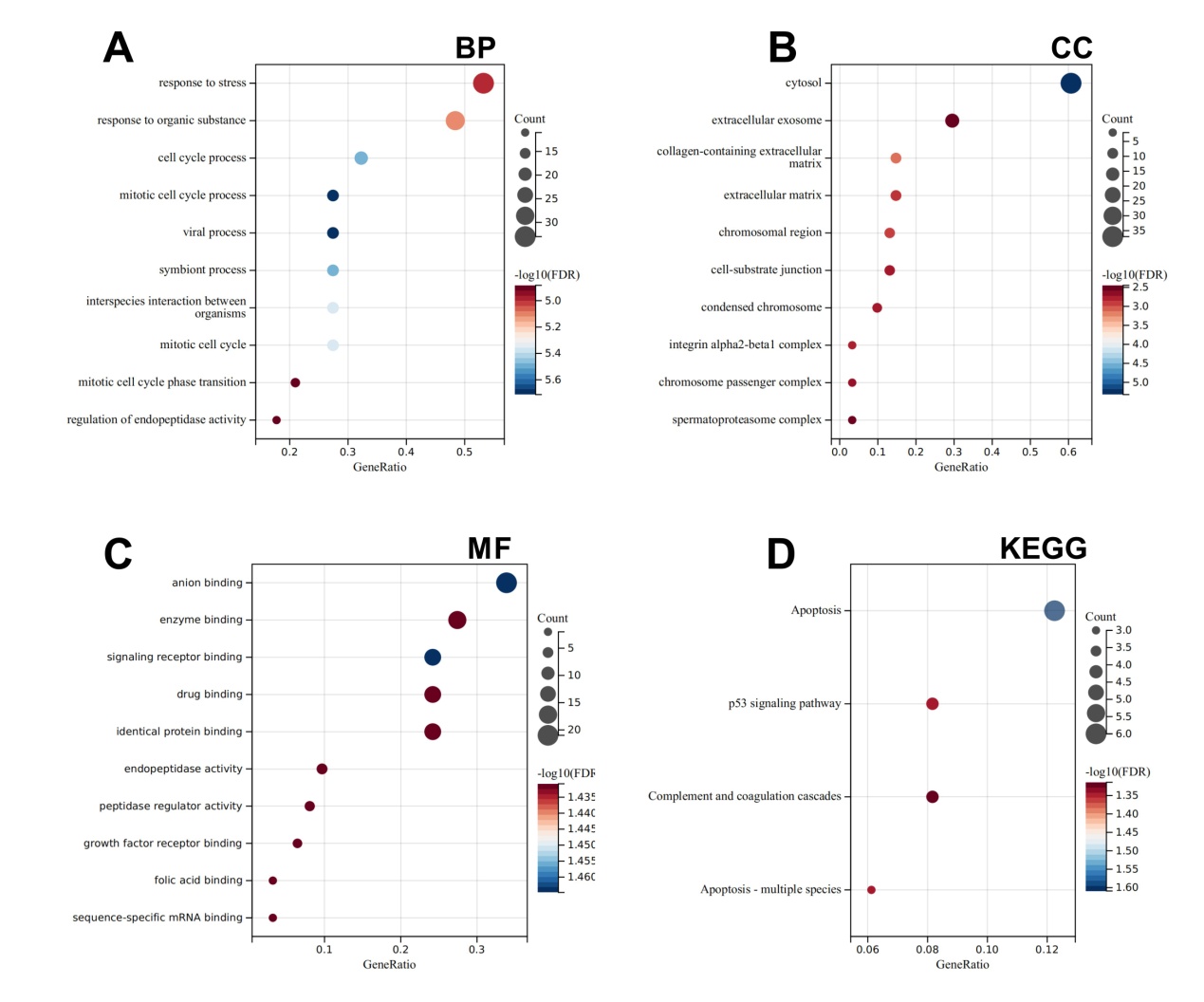


Supplementary Figure 5. Diagnostic performance of three mast cell-related hub genes based on receiver operating characteristic (ROC) analysis. (A-C) ROC curves were plotted to evaluate the diagnostic accuracy of FOS, RAD51, and RRM2, respectively, using the combined expression matrix from merged datasets. The area under the curve (AUC) was calculated to quantify the sensitivity and specificity of each gene in distinguishing disease from control samples.
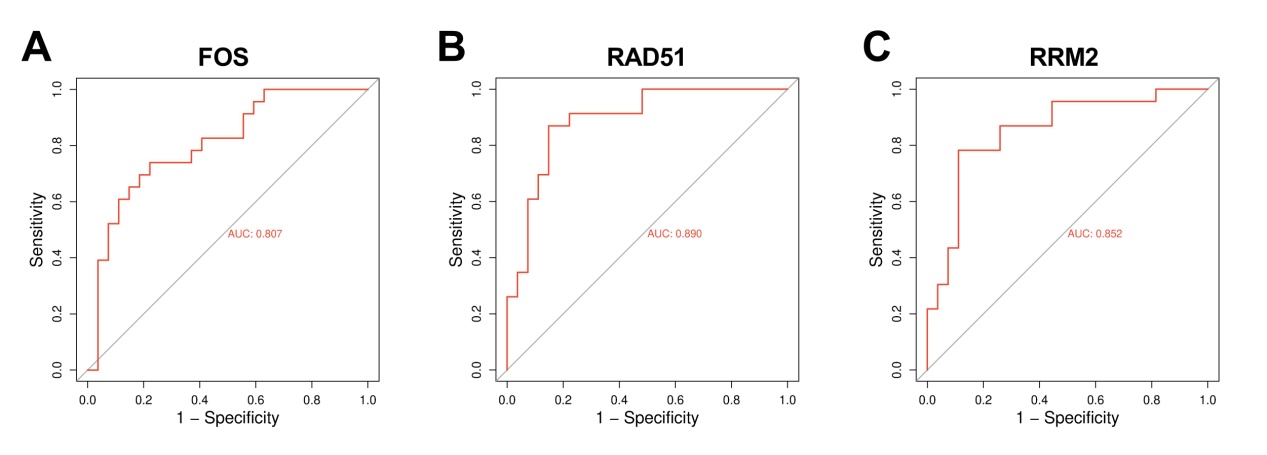

Supplement: Supplementary file 3 — Supporting Information 3 Supporting Figure 1. Data preprocessing and dimensionality reduction of merged Gene Expression Omnibus (GEO) datasets. (A) Density plots before (left) and after (right) normalization across three GEO datasets (GSE5081, GSE27411, and GSE233973), showing the distribution of gene expression values. (B) Uniform Manifold Approximation and Projection (UMAP) analysis of the three normalized datasets. (C) Principal component analysis (PCA) of gastritis and control samples, visualized with 95% confidence ellipses. (D) Three‐dimensional PCA plot showing the separation between the gastritis and normal control (NC) groups based on PC1, PC2, and PC3. Supporting Figure 2. Weighted gene co‐expression network analysis (WGCNA) for module identification and correlation. (A‐B) Determination of the soft‐thresholding power. Scale‐free topology fit index (A) and mean connectivity (B) were plotted to select an appropriate soft‐threshold. (C) Dendrogram of genes clustered based on a topological overlap matrix (TOM), with corresponding module colors assigned using dynamic tree cut and subsequent merging. (D) A dendrogram of module eigengenes and an adjacency heatmap were constructed to illustrate inter‐module similarity. (E) Module–trait relationship analysis illustrating correlations between module eigengenes and clinical traits. Correlation coefficients are shown within each cell, with color intensity reflecting the strength and direction of the association. (F) Scatter plot of gene significance versus module membership in the selected key module. Supporting Figure 3. Identification and interaction analysis of mast cell‐related DEGs. (A) Venn diagram showing the intersection among DEGs, WGCNA‐derived hub module genes, and mast cell‐related genes obtained from the GeneCards database. (B) Protein–protein interaction (PPI) network of the intersected mast cell‐related DEGs constructed using the STRING database and visualized in Cytoscape. (C) Identification of hub gen [file MI-2026-4596288-s002.docx]
